# Maleic acid as an important monomer in synthesis of stimuli-responsive poly(acrylic acid-co-acrylamide-co-maleic acid) superabsorbent polymer

**DOI:** 10.1038/s41598-023-30558-3

**Published:** 2023-03-02

**Authors:** Fatemeh Jamali, Negar Etminani-Esfahani, Abbas Rahmati

**Affiliations:** Department of Chemistry, University of Isfahan, P.O. Box 81746-73441, Isfahan, Iran

**Keywords:** Environmental sciences, Chemistry, Engineering, Materials science

## Abstract

Poly(acrylic acid-co-acrylamide-co-maleic acid) (p(AA-co-AM-co-MA)) superabsorbent polymer was synthesized from acrylic acid (AA), acrylamide (AM), and maleic acid (MA) via free radical copolymerization. Results showed the presence of maleic acid in structure of superabsorbent has the key and superior role in creating a smart superabsorbent. The structure, morphology, and strength of the superabsorbent were characterized using FT-IR, TGA, SEM, and rheology analysis. The effect of different factors was investigated to determine the ability of water absorbency of the superabsorbent. According to optimized conditions, the water absorbency capacity of the superabsorbent in distilled water (DW) was 1348 g/g and in a solution containing 1.0 wt.% NaCl (SCS) was 106 g/g. The water retention ability of the superabsorbent was also investigated. The kinetic swelling of superabsorbent was identified by Fickian diffusion and Schott's pseudo-second-order model. Furthermore, the reusability of superabsorbent was studied in distilled water and saline solution. The ability of superabsorbent was investigated in simulated urea and glucose solutions, and very good results were obtained. The response ability of the superabsorbent was confirmed by swelling and shrinking behavior against changes of temperature, pH, and ionic strength.

## Introduction

One of the most important industrial materials in the world is superabsorbent polymers (SAPs). Global demand for manufacturing of SAPs is very abundant because they are used in hospital bed pads, baby and adult diapers^1^ as well as agriculture^2^, food processing^3^, water/sewage treatment^3^, tissue engineering^4^, sensors^5^, and drug delivery^6^. SAPs are a type of hydrophilic hydrogels that can absorb and hold massive amounts of water or other aqueous solutions up to hundreds of times their dry weight. These three-dimensional polymeric networks are not dissolved in water and physiological solutions owing to chemical or physical crosslinks^7^. Although a great deal of attention has been paid to the production of superabsorbents using natural materials, it has not yet been industrialized due to the high price of these materials. Still, AA and AM are the best monomers in the industry to synthesize superabsorbents due to easy production, low-cost, availability and rapid polymerization^8,9^. AA and AM have high hydrophilicity, excellent flexibility, chelating properties, and biodegradablity^10^. Moreover, it was found that copolymerization of AA and AM in a polymeric network can improve the strength of the gel^11^. On the other hand, maleic acid (MA) is the other industrially synthetic compound that has lately gained popularity in the production of polymers due to its lower cost, good compatibility, and nontoxicity^12,13^. Thus, preparation of a novel superabsorbent with these cheap molecules and worthwhile properties is very vital.

Sensitive hydrogels have attracted much attention due to absorbing/releasing materials and swelling/deswelling behavior in response to physical, chemical and biological stimuli^14–16^. Among them, temperature, pH, and ionic strength are very important owing to simplicity and no requirement for complex devices and expensive materials^17^. In regard to the preparation of temperature-sensitive hydrogels, it has been proven that the presence of hydrophilic and hydrophobic functional groups plays a decisive role^18^. When hydrophilic monomers are copolymerized with *N*-isopropylacrylamide (having isopropyl hydrophobic part) create a temperature-sensitive hydrogel^19^. Also, chitosan, cellulose, gelatin, and poloxamers and their derivatives are suitable starting materials for the synthesis of thermo-responsive hydrogels^20^. On the other hand, it has been specified the presence of anionic and cationic functional groups has a vital role in the synthesis of pH-sensitive hydrogels^21–23^. When acrylic acid derivatives react with natural polymers such as carboxy methyl dextran, albumin, gelatin, alginate, and chitosan are synthesized pH-responsive hydrogels^21–23^. Thus, to find a new monomer structure in production thermo- and pH-sensitive property in preparation of smart hydrogels can be very important. There is no smart superabsorbent from AA, AM and MA monomers.

**Scheme 1:**
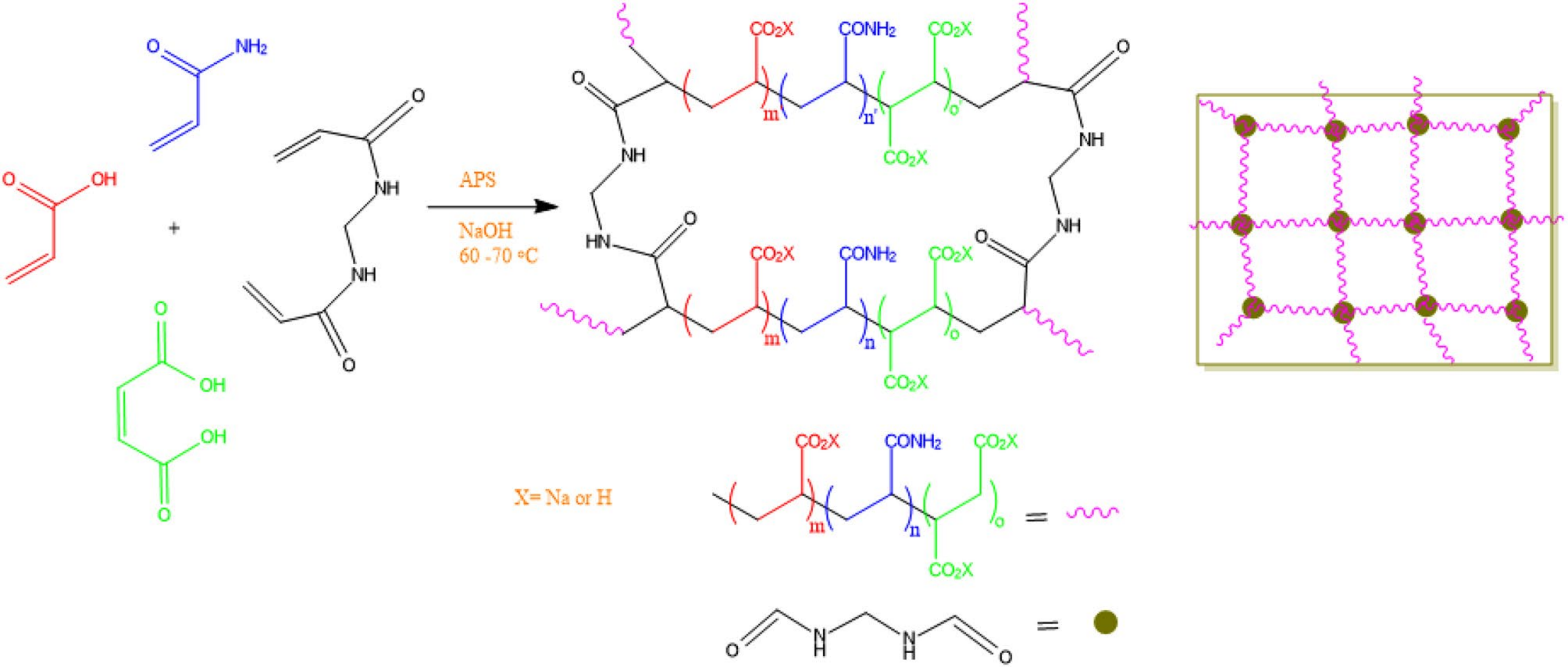
Synthesis of p (AA-co-AM-co-MA) and its suggested structure.

Thus in this article, not only the synthesis of a worthy superabsorbent using cheap and available starting materials is described (Scheme 1), but also MA is introduced as a vital monomer in the preparation of intelligent superabsorbent which can respond to various stimuli such as temperature, pH and ionic strength media.

## Materials and methods

### Materials

*N,N'*-methylenebisacrylamide (98%) (MBA), acrylamide (99.9%), maleic acid (99%), acrylic acid (99%), sodium hydroxide, and ammonium persulfate (98%) (APS) were provided in analytical grade from Merck.

### Synthesis of p(AA-co-AM-co-MA) Superabsorbent

To prepare the smart superabsorbent (p(AA-co-AM-co-MA)), AA (1.80 g, 25 mmol), AM (0.73 g, 10 mmol), and MA (1.47 g, 12 mmol) were completely dissolved in 13 mL DW in the beaker using a magnetic stirrer. Subsequently, NaOH (2.00 g, 50 mmol) and MBA (0.0045 g, 0.029 mmol) were added to the reaction mixture. The solution was then allowed to heat above 60 °C. In the following, (0.018 g, 0.079 mmol) of APS was poured into the previous solution, and the polymerization reaction was done for 2 h. Then, the prepared superabsorbent was dried at room temperature. Then, the obtained superabsorbent was immersed in DW to remove homopolymers and unreacted materials. Finally, the obtained superabsorbent was dried in oven at 60 °C.

All the reactions of the optimization stages were performed in the same way but with different amounts of AM, AM, MA, APS, NaOH, and MBA. In addition, the p(AA-co-AM) superabsorbent was prepared with this method without using the MA monomer.

### Characterization techniques

FT-IR spectra were obtained at room temperature using the KBr disk method with a JASCO FT/IR-6300 spectrometer to identify functional groups in polymeric chains. Rheological characterization was done to determine the strength superabsorbent by Anton-Paar Physica MCR 301 machinery. Thermal stability was evaluated by the thermogravimetric analysis (TGA-Perkin Elmer) from 25 to 800 °C with a heating rate of 10 °C/min under N_2_ flow. A DME Dualscop C-26 microscope was used to study the morphology of the superabsorbent. A Metrohm pH meter (827 pH lab) was used to control pH of solutions. To perform these analyses (FT-IR, Rheology, TGA, and FE-SEM), an FD-5005-BT freeze dryer was used to lyophilize the superabsorbents, p(AA-co-AM-co-MA) and p(AA-co-AM), at − 75 °C for 48 h.

### Measurement methods

Three equal samples were used for calculations, and the results were expressed as an average.

#### The capacity of water absorbency

A certain amount of the superabsorbent was immersed for 24 h in various solutions (including different pH solutions and various kinds of salt solutions) at 25 °C. The swollen superabsorbent was then taken out from the solution, and the capacity of the water absorbency was calculated according to Eq. (1).^24^

1 $$\mathrm{Q}=\frac{{\mathrm{W}}_{1 }{-\mathrm{ W}}_{0}}{{\mathrm{W}}_{0}}$$

In this equation, Q (g/g), W_1_ and W_0_ are the capacity of water absorbency, the swollen and dry weights of the superabsorbent, respectively.

#### Swelling and shrinking

Swelling/shrinking behavior of the superabsorbent was measured utilizing the typical gravimetric method. At first, the dried sample was swelled in aqueous media with clear features and special conditions (in terms of ionic concentration, pH, and temperature). Next, the swollen superabsorbent was separated from the medium and weighted at certain times. Subsequently, to observation of the amount of shrinking, the swollen superabsorbent was put in another medium with different features and conditions. Then, its weight was measured as long as it was swollen. These swelling and shrinking investigations were performed for three cycles.

#### Water absorbency kinetics

To determine the kinetic of water absorbency, one gram of the superabsorbent was soaked in excess DW or SCS and calculated the amount of Q. These kinetic data were recorded at the equal time distances. In the subsequent studies, a Fickian diffusion model (Eq. 2) and a pseudo-second-order kinetic model (Eq. 3) were applied to fitting the Q amounts at different times (t)^9,24^.

2 $$f=\frac{{\mathrm{Q}}_{\mathrm{t }}}{{\mathrm{Q}}_{\mathrm{e}}} =\mathrm{K}{t}^{n}$$

3 $$\frac{t}{{\mathrm{Q}}_{\mathrm{t }}}=\frac{{1}_{ }}{{\mathrm{K}}_{2}{{Q}_{e}^{2}}_{ }}+ \frac{t}{{\mathrm{Q}}_{\mathrm{e}}}$$

In the above equation, the water absorbency of the superabsorbent at time t and after it becomes equilibrium are Q_t_ (g/g) and Q_e_ (g/g), respectively. In Eq. (2), K and n correspond to the characteristic constant and the diffusional exponent of the superabsorbent, respectively. K_2_ is the rate constant of swelling in Eq. (3).

#### Water retention studies

To find the amount of the superabsorbent water retention, the samples were weighed upon equilibration (Q_e_) in DW or SCS. In the following step, the samples were placed in oven at various temperatures of 25 and 50 °C. After that, each sample was weighed once every hour (Q_t_) for nine hours. The water retention (W_r_) was calculated by Eq. (4)^25^.

4 $${W}_{r}=\frac{{\mathrm{Q}}_{\mathrm{t }}}{{\mathrm{Q}}_{\mathrm{e}}}$$

## Results and discussions

### To optimize condition for the synthesis of the superabsorbent

**Figure 1:**
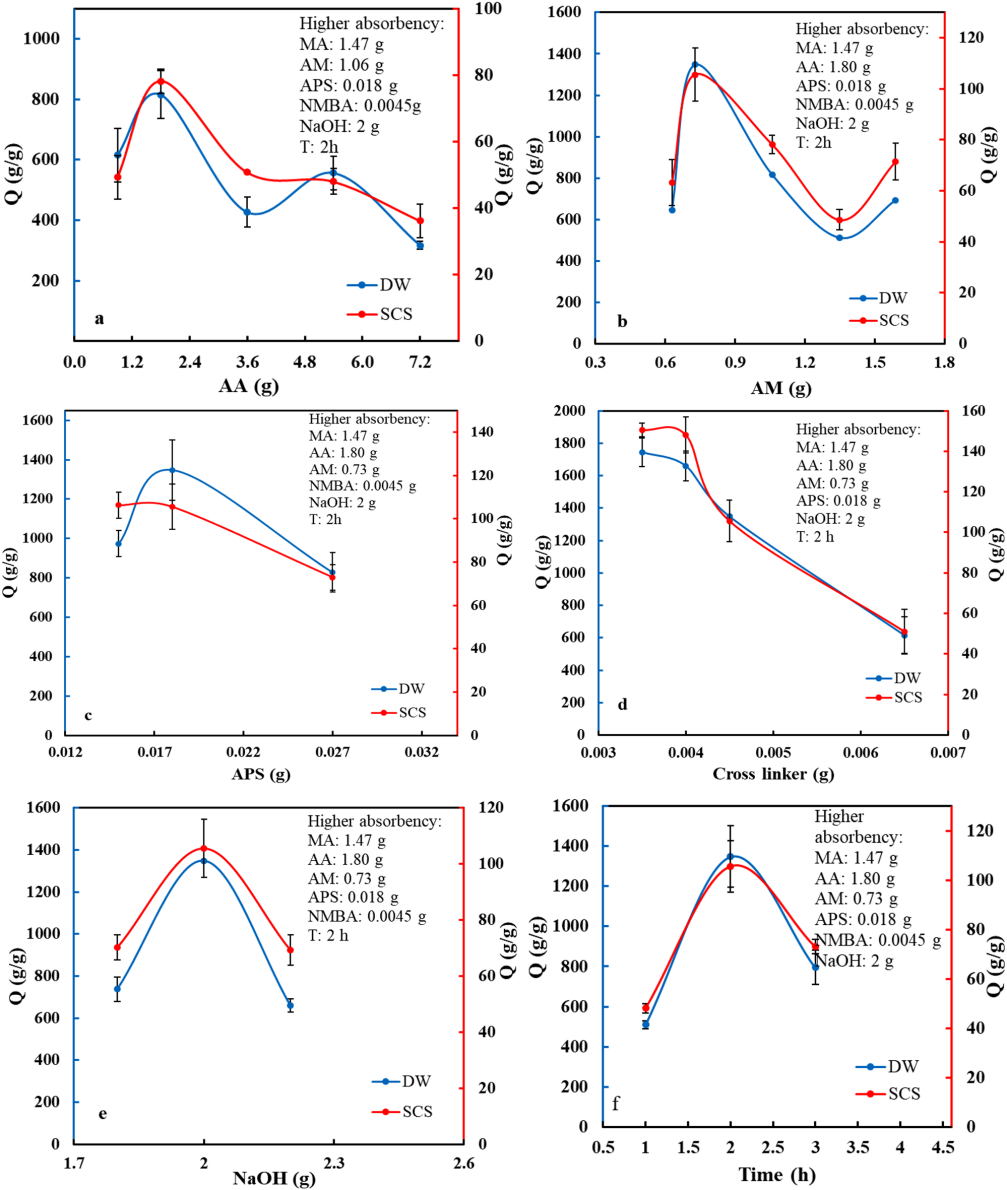
The water absorbency capacity of the p(AA-co-AM-co-MA) superabsorbent under the effect of the following variables: (**a**) Amount of AA. (**b**) Amount of AM. (**c**) Amount of APS initiator. (**d**) Amount of MBA cross-linker. (**e**) Neutralization amount. (**f**) Reaction time. Data for DW (blue color), Data for SCS (red color).

To find a superior condition in the synthesis of a p(AA-co-AM-co-MA) superabsorbent with maximum water absorbency various parameters were considered in DS or SCS. To find the desired amount of starting materials, the amounts of AA, AM, MA, APS, NaOH, and NMBA were changed. When obtaining the optimal amount of one substance, the amounts of the other materials were kept constant. The results of various parameters are as follows:

Varying the amount of AA monomer on the ability of the water absorbency was investigated (Fig. 1a). It was found that the maximum water absorbency capacity in DW was 816 ± 79 g/g, and in a SCS was 78 ± 4 g/g with amount 1.80 g of AA monomer. At first, the amount of water absorbency is increased with the addition of AA content. Acrylic acid is a monomer that causes superabsorbent flexibility. With the increase in flexibility, the amount of water absorption by superabsorbent increases. The water absorbency capacity decreases after reaching maximum, because by increasing the concentrations of AA homopolymerization was acquired with a greater rate and the rest of the molecules do not participate in the reaction^26^. Thus, synergic effects of other functional groups has been reduced. After that by addition AA the portion of MA immediately declines and in 3.6 g from AA, the role of MA is completely omitted. Subsequently, polymerization is happened between AA and AM, and by increasing the number of AA again by addition of flexibility along the chains the amount of water absorption is improved up to 556 g/g water absorbency with 5.67 g AA (the second maximum water absorbency). After this, water absorbency reduces again owing to AA homopolymerization and creation of cross-linkers between acid groups.

AM as a non-ionic monomer was used and optimized because it has excellent salt resistance feature^27^. Fig. 1b shows the effect of AM amounts on water absorbency capacity. The results showed that the maximum absorbency capacity reached 1348 ± 153 g/g in DS and in SCS gained 106 ± 10 g/g with 0.73 g AM. The initial increase up to the first maximum is due to the hydrophilic nature of AM monomer. After maximum, similar to the increasing AA, the portion of MA (that is the main material in absorption factor) reduces by addition of AM. When the amount of AM arrived to 1.35 g, MA completely remove. Then, the amount of water absorbency increases again by accumulative the amount of AM, which is due to the hydrophilicity of amide groups. But the increase did not continue. Dropping water absorbency after 1.59 is owing to increasing medium viscosity which prevents the movement of free radicals and active species of reactants in the medium^28^.

Changes in the concentration of MA showed that this compound with 1.47 g has the maximum amount of water absorption. But in lower than this amount the water absorbency was decreased and higher than this the value of water absorbency is constant.

Changing the amounts of APS on the amount of water absorbency has been shown in Fig. 1c. Results directed the maximum water absorbency capacity in DS is 1348 ± 153 g/g, and in SCS is 106 ± 10 g/g with amount 0.018 g of APS. According to Allcock and Frederick studies, the increased amount of the initiator decreases the molecular weight of the polymer in free radical polymerization. This decrease in the molecular weight causes an increase in polymer chain ends^25^. It seems that with increasing initiator content, the cross-linked density enhances, which affects the capacity of water absorbency. The capacity of water absorbency by the superabsorbent was decreased with the initiator content lower than 0.45 wt.%. Possibly this is due to the reduced content of free radicals generated by the initiator, so the network cannot be effectively formed^29^.

According to the Flory's network theory, cross-linker is a significant factor that affects network density and the water absorbency capacity of the superabsorbent. In this theory, the increasing cross-linkers raise the network nodes and density^30^. Consequently, the more amount of cross-linker increases the rigidity of the structure and limits the amount of water absorbency^30^. According to Fig. 1d, the maximum absorbency in DS is 1744 ± 87 g/g with the cross-linker amount in the range of 0.0030–0.0040 g. However, this amount was not selected as the optimum amount because the dimensional structure of the superabsorbent was unstable, and the mechanical property was negligible. With amounts, lower than 0.0030 g is only created viscous gel. According to the mechanical strength obtained from the rheology analysis, the fabricated superabsorbent with 0.0045 g of cross-linker was selected as the optimum amount. The water absorbency capacity by this amount in DW was 1348 ± 153 g/g, and in SCS was 106 ± 10 g/g.

The degree of neutralization is an important parameter of the polymerization rate and three-dimensional network charge density. As a result, it considerably affects the amount of water that can be absorbed by the superabsorbent. The neutralization degree was defined as the molar percentage of carboxyl groups in AA and MA neutralized by NaOH^29^. The maximum capacity of water absorbency is shown in Fig. 1e with 2 g neutralization which was 1348 ± 153 g/g and 106 ± 10 g/g in DW and SCS, respectively. The capacity to absorb water enhances with an increasing amount of neutralization until 2 g, due to the more active carboxylate groups of the AA and MA. It can be attributed to the electrostatic forces repulsion between carboxylate groups that are created by the neutralization of acrylic acid and the number of osmotically active ionic carboxylate groups in the copolymer^31^. When the neutralization degree is more than 2 g, the water absorbency capacity is decreased. It may be due to hydrolysis of cross-linkers and the increasing solubility of the product owing to the further number of carboxylate groups^29^.

As shown in Fig. 1f, the effect of reaction time was studied on the water absorbency capacity of the superabsorbent. At 2 h, the maximum capacity in DW was 1348 ± 153 g/g, and SCS was 106 ± 10 g/g. In shorter times polymerization is presumably incomplete, thus causing to increase the solubility of the polymer fraction and insufficient formation of cross-linkers. Longer reaction times lead to a high number of cross-linkers in the polymer network, which inhibits the stretching of the polymer chains in the network and reduces the capacity of water absorbency^29,32^.

According to the obtained results, the best conditions for the synthesis of p(AA-co-AM-co-MA) superabsorbent, with maximum water absorbency capacity includes AA (1.80 g), AM (0.73 g), MA (1.47 g), and NaOH (2 g), 2 h reaction time, and 60–70 °C reaction temperature. To prove the effect of MA, the p(AA-co-AM) superabsorbent was synthesized under the optimum condition without MA, and its water absorbency capacity was also measured (866 ± 41 g/g). Thus, results confirmed the efficiency of malic acid in the structure of the p(AA-co-AM-co-MA) superabsorbent.

### Characterizations

**Figure 2:**
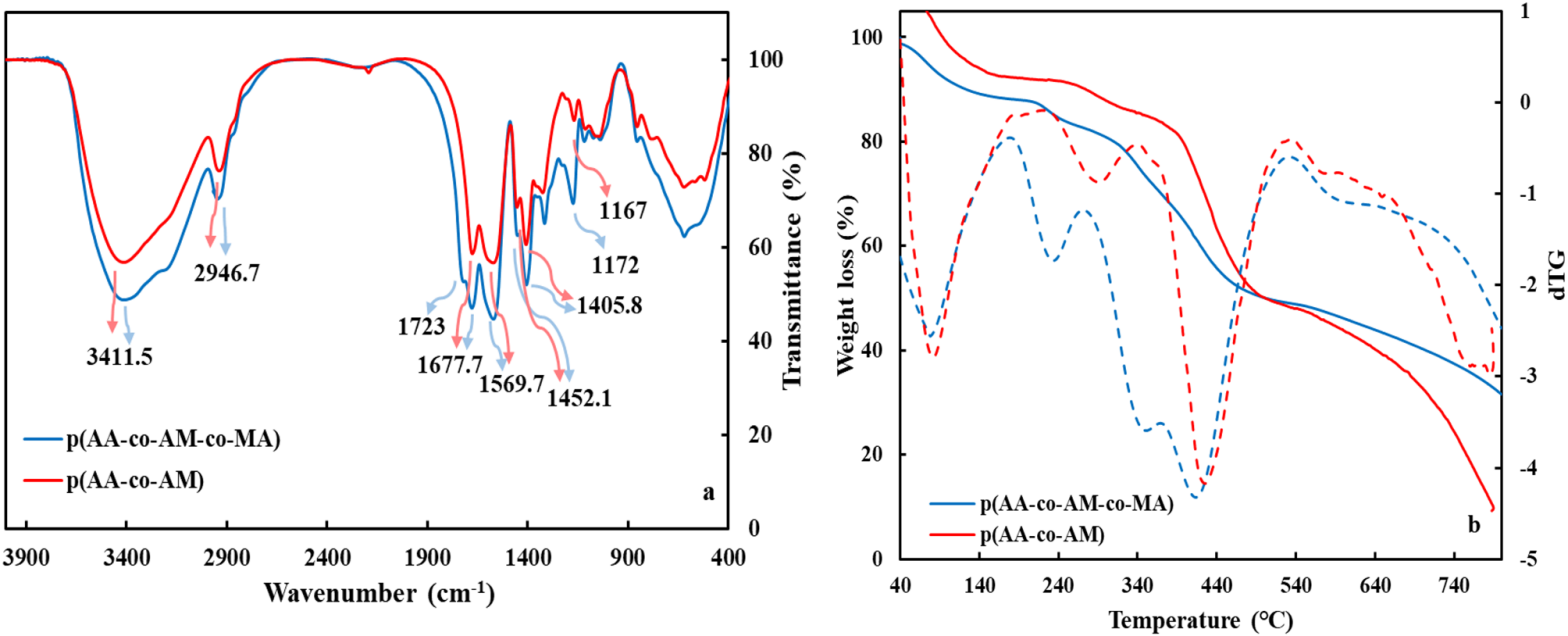
(**a**) FT-IR spectra of p(AA-co-AM-co-MA) and p(AA-co-AM), and (**b**) TGA and dTG of p(AA-co-AM-co-MA) and p(AA-co-AM) superabsorbents.

FT-IR spectrum of the p(AA-co-AM-co-MA) superabsorbent is shown in Fig. 2a. The broadband at 3411.5 cm^−1^ is ascribed to the stretching vibration of the NH group of the AM unit. Also, it has overlapped with the stretching vibration of the –OH group of carboxylate units^33^. The band at about 2946.7 cm^−1^ is related to the C–H absorption band of the methylene groups of p(AA-co-AM-co-MA). The absorption bands at 1723 cm^−1^ and 1677.7 cm^−1^ are assigned to the C=O stretching vibrations of carboxyl and amide groups, respectively^34,35^. Also, –N–H bending of the amide has overlapped with C=O of amide groups. The band at 1452.1 cm^−1^ is attributed to the symmetric stretching −CH=CHCOO^−^Na^+^ and asymmetric stretching vibrations of groups COO^−^ is at 1569.7 cm^−1^^36^. In addition, the bands at 1405.8 cm^−1^ and 1172.5 cm^−1^ correspond to the C–N and C–O stretching of the –CONH_2_ and COOH units, respectively^37,38^. The FT-IR results clearly proved that the p(AA-co-AM-co-MA) superabsorbent was successfully prepared. Also, the comparison of changes in the peak intensities of the two superabsorbents indicates the successful copolymerization of MA in the structure of the polymer network.

Figure 2b shows the TGA and DTG curves of the p(AA-co-AM-co-MA) and p(AA-co-AM) superabsorbents. The curves of the p(AA-co-AM-co-MA) superabsorbent show a Five-step decomposition. The first step of weight loss is at around 40–170 °C because of water loss, which is absorbed on the surface and in the network pores. The second step was observed in the range of 175 to 290 °C, which is due to the water elimination from two carboxyl groups of the MA units and cyclic anhydride formation^39^. In the third step, range of 260 to 384 °C, the amide groups of AM units and the cross-linkers on the polymer chains were decomposed. The fourth step, between 394 to 520 °C, is owing to remove of carboxylate and anhydride groups on the polymer chain. The final step (above 520 °C) is the breaking of the main polymer chains and the destruction of the polymer network^40–42^. While, the curves of the p(AA-co-AM) superabsorbent show a four-step decomposition. The first step weight reducing similar to the p(AA-co-AM-co-MA) superabsorbent is at range 50–218 °C because of surface and network water loss. The second step in the range of 230 to 352 °C, the amide groups of AM units and the cross-linkers are removed from the polymer chains. The third step, in the range of 360 to 537 °C, is related to the destruction of the carboxyl groups of AA units from the polymer chain. The fourth step of decomposition, above 540 °C is ascribed to the breakdown of the main chain and the organic residues^40–42^. Therefore, TGA and DTG analysis confirm that p(AA-co-AM) superabsorbent has one stage less than p(AA-co-AM-co-MA) superabsorbent, this can be attributed to the presence of maleic acid.

**Figure 3:**
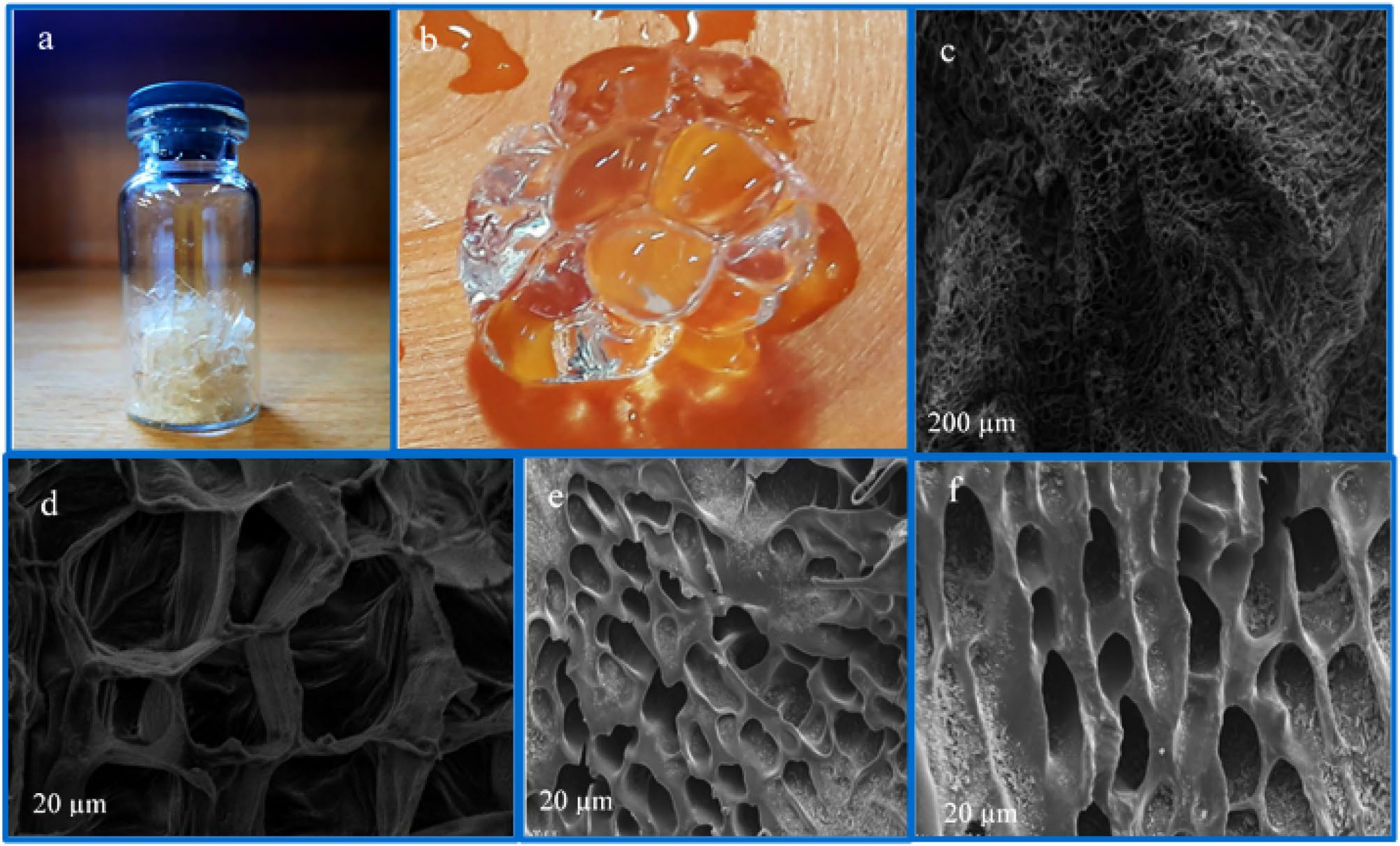
Digital photos of p(AA-co-AM-co-MA) in dry (**a**) and swollen modes (**b**), respectively. SEM micrographs of p(AA-co-AM-co-MA) superabsorbent (**c**,**d**), and SEM micrographs of p(AA-co-AM) superabsorbent (**e**,**f**).

The appearance of p(AA-co-AM-co-MA) superabsorbent in dried and swollen states is shown in Fig. 3a,b. There was a considerable difference between dried and swollen superabsorbents. The form of swollen superabsorbents is completely spherical contrary to flat form of the dried superabsorbent.

Figure 3c,d shows the SEM micrograph of the superabsorbent. The structure of the prepared superabsorbent from AA, AM, and MA, due to the presence of the large interconnected pores, provided excellent surface and absorption functions^43^. Also, these interconnected pores are in the form of large hollow channels that can facilitate the rapid movement of water or other solvents. Also, SEM micrographs of the p(AA-co-AM) superabsorbent are presented in Fig. 3e,f. These indicate the structure and size of pores are different relative to the surface of the p(AA-co-AM-co-MA) superabsorbent. This can be attributed to the participation of the MA monomer.

**Figure 4:**
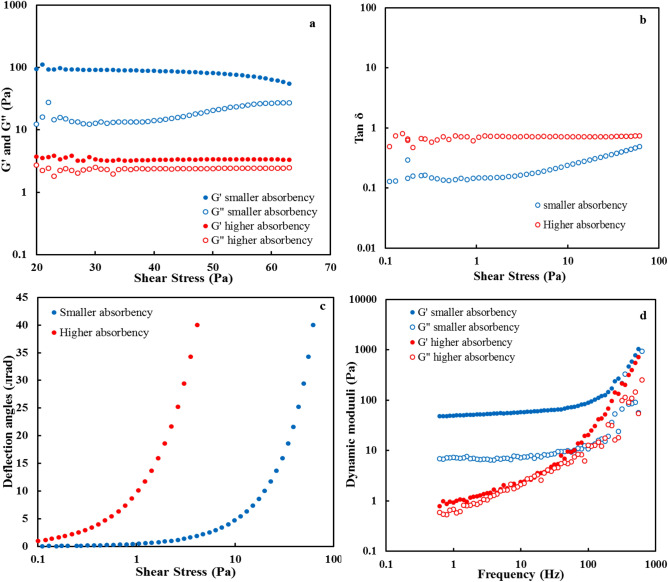
Viscoelastic properties of the superabsorbents swollen in DW: (**a**) G' and G" as a function of shear stress, (**b**) tan δ versus shear stress, (**c**) deflection angle versus shear stress, and (**d**) G' and G" versus frequency for the swelling superabsorbents.

Rheological analysis was used to study the viscoelastic properties of the prepared superabsorbents. For this aim, the linear viscoelastic range of the superabsorbent, LVE (linear viscoelastic), was first determined through stress sweep tests. In this range, the storage modulus (G′), and the loss modulus (G″) do not depend on applied stress (Fig. 4a). Also, this Figure shows that the superabsorbent with smaller absorbency (1347.72 ± 153.09 g/g in DW) has greater critical stress (σc) than the superabsorbent with higher absorbency (1743.59 ± 86.83 g/g in DW). This indicates its greater resistance to external stresses. Another significant rheological parameter in determining the strength of the gel is the damping coefficient (tan δ). Figure 4b shows the plot of the shear stress-damping coefficient. An increase in tan δ is indicative of liquid behavior (tan δ greater than 1), while a decrease in the value indicates solid-like (gel) behavior (tan δ less than 1)^44^. Smaller tan δ values indicate stronger interactions within the polymer system^45^. As shown in Fig. 4b, the tan δ value of the superabsorbent with high absorbency was rather close to 1, indicating that it had a more liquid-like behavior (less gel strength). Therefore, it showed that water absorbency capacity and gel strength are inversely related^44^, as well as the phase angles of two superabsorbents, were investigated. The phase angle of a perfectly elastic material is 0^°^ (0 π rad), and that of a perfectly viscous material is 90^°^ (π/2 rad)^46^. It can be seen from the results that the deflection angle of the superabsorbent with less absorbency (1347.72 ± 153.09 g/g) in a range of stresses (0.1–1) is close to that of fully elastic material (Fig. 4c). In contrast, the higher absorbency superabsorbent is close to the viscous material.

Subsequent to the stress sweep experiments, the frequency sweep tests were accomplished in the range of 0.1 to 1000 rad/s. Figure 4d shows the results of these experiments. G′ values of the superabsorbent with smaller absorbency were significantly higher than G″ values at all frequencies, indicating more solid-like (gel-like) behavior. Thus, the gel of this superabsorbent with smaller absorbency had greater strength than the gel of the superabsorbent with higher absorbency capacity^44^. In gels of the superabsorbent with smaller absorbency, G′ is greater than G″, showing that elastic behavior overcomes viscoelastic behavior. In addition, the samples tend to be more rigid at higher frequencies because their flexibility is reduced^47^.

### Measurement

#### Kinetic studies

**Figure 5:**
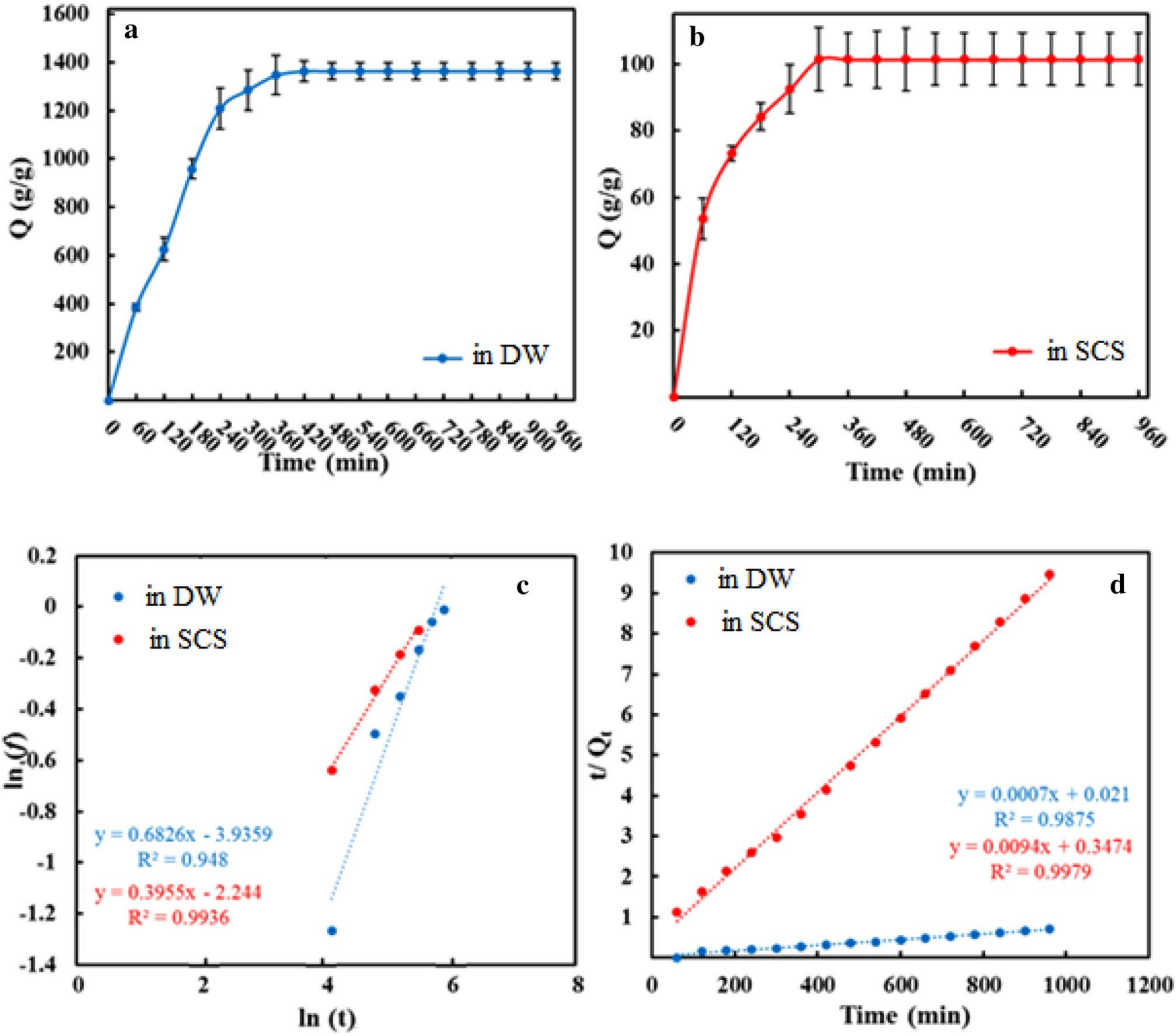
Swelling kinetics of p(AA-co-AM-co-MA) superabsorbent (**a**,**b**). Two classic kinetic theories models are studied (**c**) The ln t dependence of ln f and (**d**) relationships of t/Q_t_ with t for p(AA-co-AM-co-MA) superabsorbent.

As shown in Fig. 5a,b, swell kinetics curves in DW and SCS for the p(AA-co-AM-co-MA) superabsorbent are similar. As depicted, in both DW and SCS, the water absorbency rises quickly in the initial steps and gradually increases to reach equilibrium. This swelling behavior can be explained by variations in osmotic pressure differences. In the structure of the p(AA-co-AM-co-MA) superabsorbent, the osmotic pressure difference is initially significant because of the presence of hydrophilic groups as well as electrostatic repulsion between carboxylate functional groups (anionic groups). Consequently, the water molecules can easily penetrate the superabsorbent polymer network, which increases the swelling rate. Nevertheless, the osmotic pressure difference is reduced by increasing water molecules in the network. Thus, the swelling rate gradually slows down and reaches equilibrium^48^.

The Fickian diffusion model was also applied to explain the mechanism of water molecules penetrating into the superabsorbent in DW and SCS during the initial step. For this aim, ln (f) versus ln (t) was plotted to find the amounts of n (slope) and k (intercept), based on Eq. (2) (Table 1). In Fig. 5c, the curves show good linear correlations. Based on the diffusion mechanism category^7,24^, the water absorbency behavior of superabsorbent in DW is governed by both Fickian diffusion and polymer chain relaxation (non-Fickian diffusion). Whereas, the water absorbency in SCS is dominant by the Fickian diffusion.

**Table 1:** Swelling kinetic parameters of p(AA-co-AM-co-MA) superabsorbent.

Sample	Fickian diffusion model			Pseudo-second order kinetic model			
	R	K	*n*	*Q*_*e, exp*_ (g/g)	R^2^	*Q*_*e, cal*_ (g/g)	K_2_ × 10^5^ (g g^−1^ min^−1^)
*In DW*							
p(AA-co-AM-co-MA)	0.95	0.02	0.68	1363.00	0.98	1428.57	2.56
*In SCS*							
p(AA-co-AM-co-MA)	0.99	0.11	0.39	101.38	0.99	111.11	27.79

Pseudo-second-order model was applied to investigate the kinetic mechanism. Using Eq. (3), the plot of t/Q_t_ against time was achieved in DS and SCS (Fig. 5d). Table 1 also lists the calculated kinetic parameters. Due to the high correlation coefficients (R^2^), in DW and SCS, the pseudo-second-order kinetic model, is suitable to describe the kinetic behavior of superabsorbent. Also, experimental results for this kinetic model were almost confirmed by using the calculated Q_e, cal_ amounts.

#### Water retention time at various temperatures

**Figure 6:**
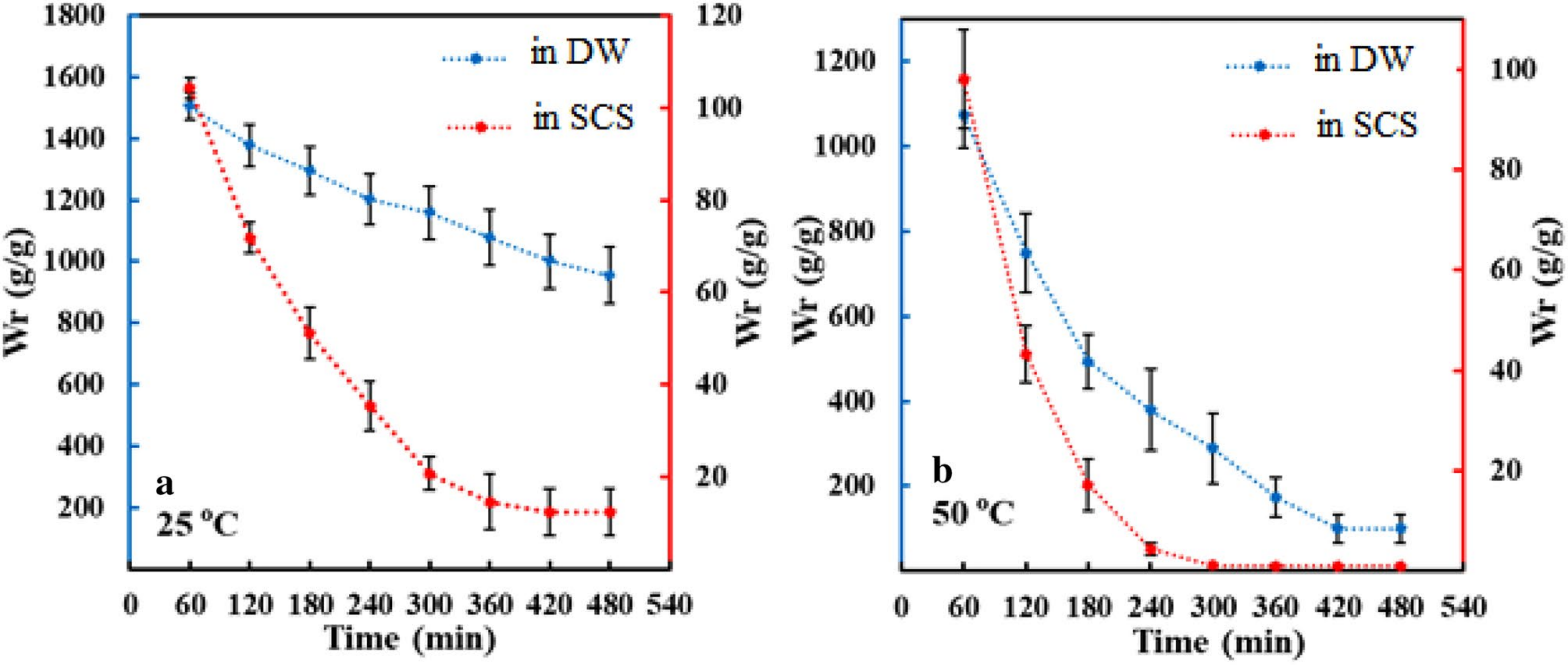
The water retention rate of p(AA-co-AM-co-MA) superabsorbent at (**a**) 25 °C and (**b**) 50 °C in DW (blue color) and SCS (red color).

In terms of the practical application of superabsorbents, measuring their water retention behavior is very important. Therefore, the water retention time of p(AA-co-AM-co-MA) in DW and SCS at various temperatures (25 and 50 °C) are shown in Fig. 6. As seen, the rate of water loss of swollen superabsorbents was different in various conditions. When the superabsorbent was placed in saline at two temperatures and in DW at 50 °C, a large amount of water was quickly lost. However, this behavior was not observed for DW at 25 °C. The results indicate that water retention of the superabsorbent is 63.54% and 9.36% in DW at 25 and 50 °C, respectively. Also, water retention in the 1wt. % NaCl solution is 11.98% and 0.96% at 25 and 50 °C, for 9 h, respectively. The water retaining can be attributed to the hydrogen bonding interaction and Van der Waals force between the water molecules and the superabsorbent^49^. It can be seen from these results that with an increasing temperature, the water retention capability decreases seriously. The cause of this behavior is probably related to the weakening of H-bonds and their breakdown at high temperatures^50^.

#### Reusability

**Figure 7:**
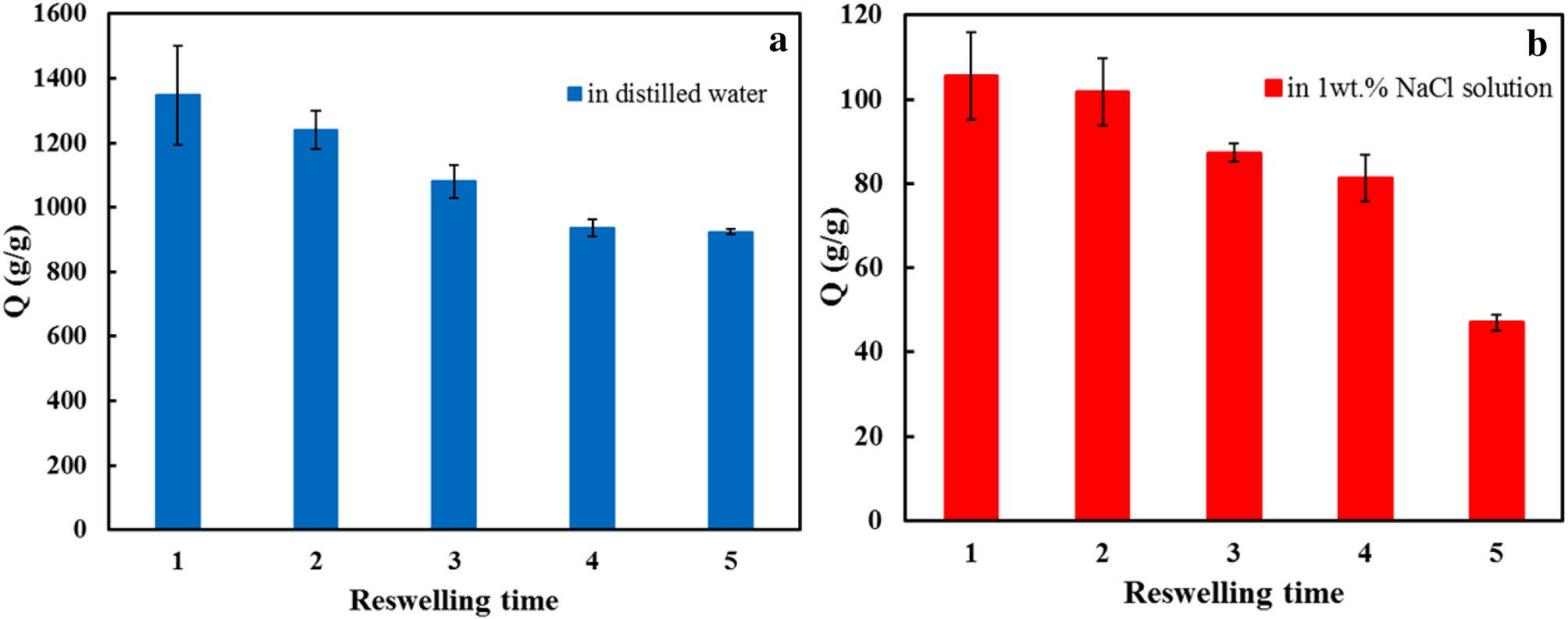
Reusability of p(AA-co-AM-co-MA) in (**a**) DW and (**b**) SCS.

The reusability and reswelling of the superabsorbent are essential factors in its efficiency. For this purpose, one gram of the superabsorbent was soaked in either DW or SCS to achieve equilibrium. The sample was then dried and used for subsequent measurements. Figure 7 shows the reusability of the p(AA-co-AM-co-MA) superabsorbent in DW and SCS. Accordingly, recycled superabsorbent was used constantly in five cycles under the same conditions. After each reusing, the water absorbency capacity of the superabsorbent a few decreases, and they can absorb 876 g/g and 48 g/g of solutions (DW and SCS) after five reswelling times, respectively. This high absorbency capacity after five cycles may be due to its stable polymeric network^51^. The breakdown of physical crosslinking in the superabsorbent structure may cause weak retention of water and ultimately decrease water absorbency capacity^52^.

## Application of superabsorbent

### Application of superabsorbent in simulated physiological fluids

**Figure 8:**
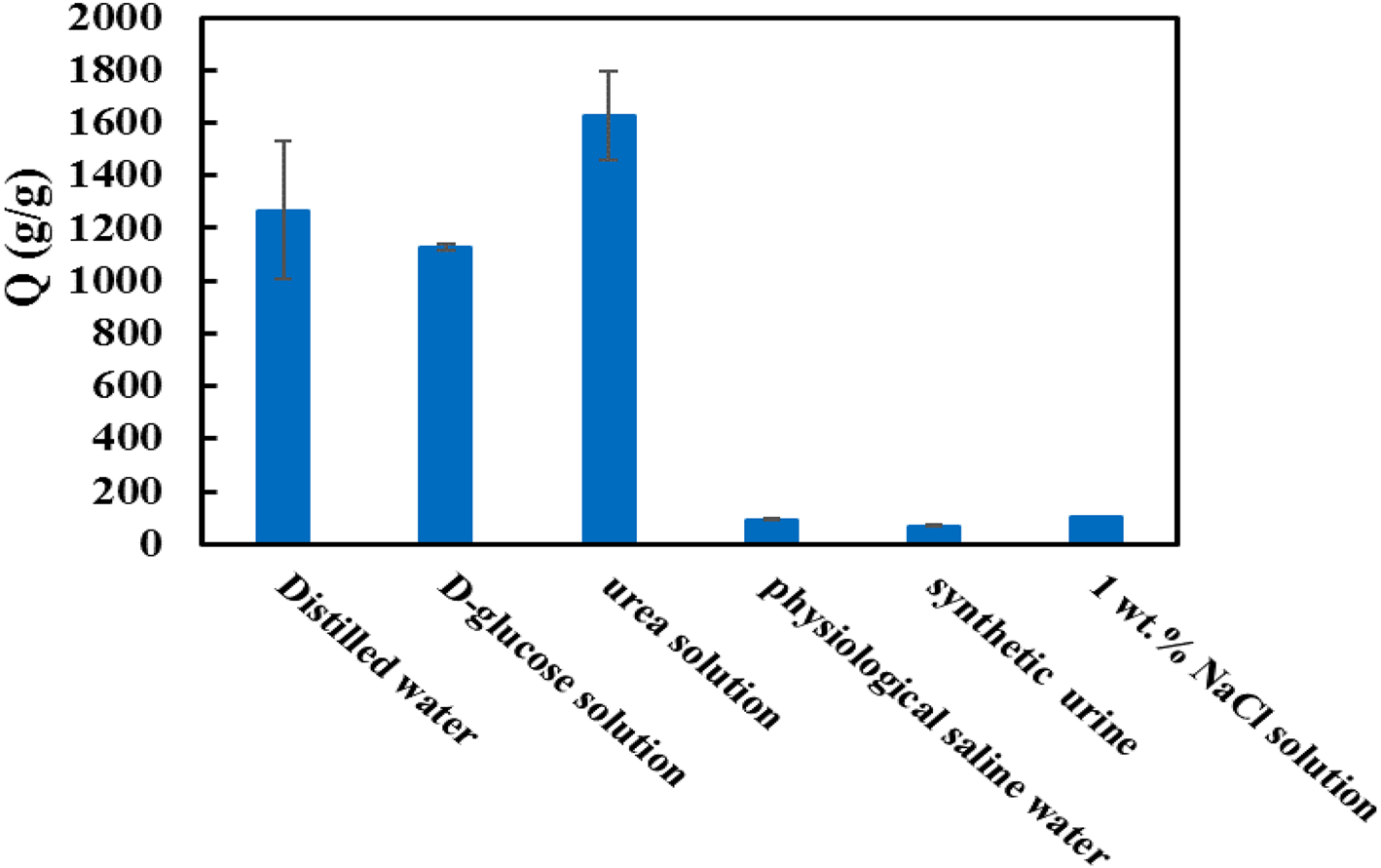
The effect of simulated biological fluids on water absorbency capacity of superabsorbent.

To expand the potential application of the prepared superabsorbent, their absorbency capacity was investigated in four kinds of simulated biological fluids, including synthetic urine, physiological saline, urea solution, and D-glucose. Among simulated physiological fluids, the capacity of superabsorbent in urea solution was greater in compared with DW medium. Also, the absorbency capacity of the superabsorbent was the same in the D-glucose solution and DW. The absorbency capacity of superabsorbent in synthetic urine and physiological saline water was reduced (Fig. 8). These results demonstrated the charge screening effect of cations (Na^+^, K^+^, Mg^2+^, and Ca^2+^) in physiological saline water and synthetic urine. This event leads to the decrease of anion–anion electrostatic repulsions (carboxylate groups on the backbones superabsorbent). As a result, the difference in osmotic pressure decrease between the polymeric network and the external medium^53^. Based on the Donnan osmotic pressure equilibrium, the more mobile cations in a solution, the lower the difference in osmotic pressure; thus, the superabsorbent shrinks^54^.

The order of the absorbency capacity of the superabsorbent in different biological fluids is as follows: urea solution > D-glucose solution > physiological saline water > synthetic urine. In addition, the absorbency capacity was more in the urea solution than the value measured in DW. In addition, the absorbency capacity of the superabsorbent in synthetic urine was similar to the physiological saline water. This phenomenon is ascribed to the presence of NaCl, which is an important contribution to the absorbency capacity of the superabsorbent^55^.

### Application of superabsorbent as a sensitive material

#### Salt-sensitivity and modulation behavior of superabsorbent

**Figure 9:**
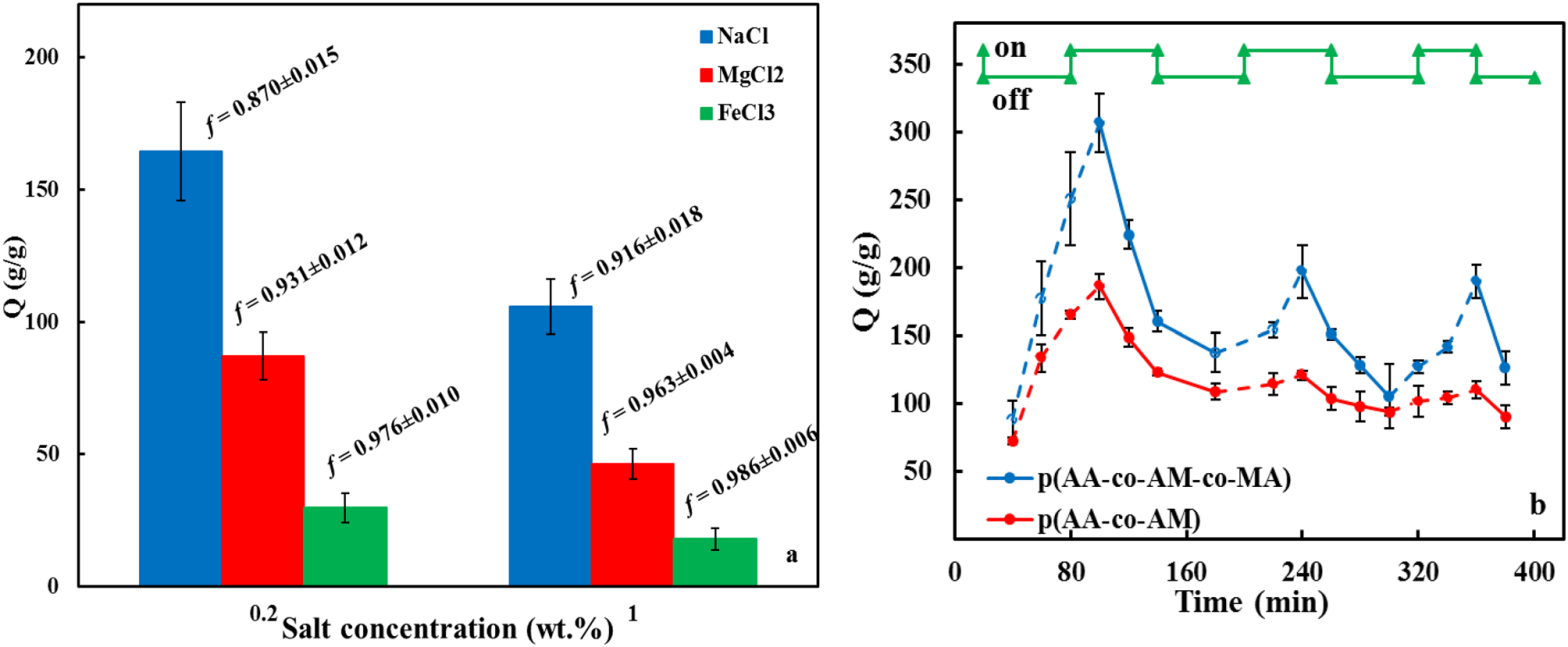
(**a**) salt sensitivity factor (*f*) and water absorbency capacity of p(AA-co-AM-co-MA) superabsorbent, (**b**) The reversible salt-controlled on–off switching for the superabsorbents in DW and SCS. The time interval between the changes was 1 h.

Figure 9a displays the ability of p(AA-co-AM-co-MA) superabsorbent to absorb water at two different concentrations of diverse salt solutions, including NaCl, MgCl_2_, and FeCl_3_. The results indicate that in salt solutions, the water absorbency capacities of superabsorbent decrease. The reason can be attributed to the protective effect of the cations in salt solutions. Salt solutions by protecting the carboxylate groups with cations prevent effective electrostatic repulsions. In this situation, the osmotic pressure difference between the polymeric network and salt solutions decreases, and as a result, the water absorbency capacity of the superabsorbent decreases. Figure 9a demonstrates the water absorbency capacity falls at upper salt concentration solutions. The phenomenon results from excess cations in solutions with higher concentrations. As well as, at an equal concentration of the salt solution, with increasing cationic charge, the capacity of the superabsorbent to absorb water decreases. The event is in accord with the theory of Flory's equation (cationic charge: univalent > divalent > trivalent)^33^.

In addition, Eq. (5) was used to calculate the dimensionless salt sensitivity factor (*f*). The *f* values of the superabsorbent for the three salts with different cations charges are shown in Fig. 9a^26^. The results show salt cations sensitivity of the superabsorbent is Na^+^ < Mg^2+^ < Fe^3+^, respectively^56^.

5 $$f =1-\frac{water \,absorption \,in \,salt \,solution}{water \,absorption \,in \,distilled \,water}$$

The modulation of the p(AA-co-AM-co-MA) and p(AA-co-AM) superabsorbents was investigated by soaking the sample in DW and SCS, respectively. The superabsorbents was swelled in DW for one hour, then soaked in SCS for one hour, and then the water absorbency capacity of the superabsorbent was determined as a function of time. Regular interval time studies show that superabsorbent swells in DW while shrinks in salt solution (Fig. 9b). This on–off switching is still present after three swelling-shrinking cycles, whereas p(AA-co-AM) superabsorbent shows a little response to ionic strength and after every time this property is decreased (Fig. 9b). By adding salt, the osmotic pressure differential between the out medium and the superabsorbent network decreases. As a result, water molecules go out of the superabsorbent and shrink^57^.

#### pH-Sensitivity behavior of superabsorbent

**Figure 10:**
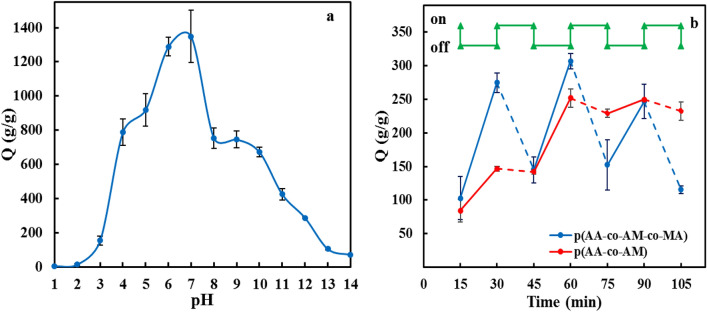
(**a**) water absorbency capacity of p(AA-co-AM-co-MA) superabsorbent at various pH solutions. (**b**) The reversible pH-controlled on–off switching for the superabsorbents in solutions with pH 6 and 3. The time interval between the pH changes was 15 min.

The sensitivity to pH was determined by analyzing the water absorbency capacities of p(AA-co-AM-co-MA) superabsorbent in different pH solutions (Fig. 10a). As the water absorbency capacity of the superabsorbent is affected by the ionic strength of the solution, buffer solutions were not utilized. Therefore, NaOH solution (alkaline pH), HCl solution (acidic pH), and DW were used to adjust the pH value of the final solution. The changes in the amount of water absorbed can be explained by the repulsion of –NH^3+^ groups in acidic media and –COO^-^ groups in basic media. With increasing the pH from 2 to 7, the water absorbency capacity increases sharply, then from 7–8 decreased and at pH 8 to 9 is almost constant, and it decreases intensely from pH 9 to 14. Water absorbency is negligible in acidic conditions due to protonation of most –COO^−^ groups and conversion to –COOH groups. Thus, anion–anion repulsive force weakens, and the network shrinks. Furthermore, hydrogen bonding interactions are enhanced due to more protonation, and the extra physical cross-linking in the polymer network is generated, leading to a decrease in water absorbency capacity^56^. As well as in acidic conditions, the charge-screening effect of the Cl^-^ counter ions shielded the ammonium cations in the polymeric network and hindered an effective repulsion^56^. With increasing pH value, the water absorbency capacities increased since the electrostatic repulsions between carboxylate groups were enhanced. Thus, the polymeric network is expanded, and water absorbency capacities achieve maximum value at pH = 6. However, most acidic groups (–COOH) and basic groups (–CONH_2_) are non-ionized forms in the pH range of 7–9. Thus, hydrogen bonds between carboxylic acid and amide groups likely cause a kind of cross-linking, and as a consequence, the capacity to absorb water decreases. In highly alkaline solutions (pH > 10.0), the water absorbency capacity decreases since the excess Na^+^ cations prevent electrostatic repulsive interactions^58^.

The modulation of the pH values in which the superabsorbent presents a shrinking/swelling state can be used for controlled drug delivery systems. As shown in Fig. 10b, the p(AA-co-AM-co-MA) swollen superabsorbent in pH 6.0 solution shrinks quickly in solution with pH 3.0, and an outstanding reversible on/off switch is achieved within 15 min. This on–off switching was done even after three swelling-shrinking cycles, but the p(AA-co-AM) superabsorbent did not respond to pH media^59^.

#### Thermo-sensitivity behavior of superabsorbent

**Figure 11:**
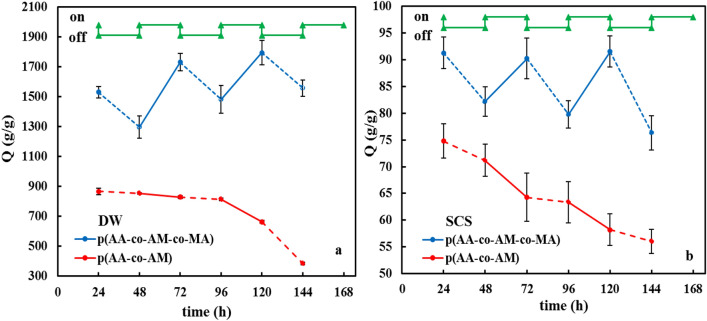
The reversible T-controlled on–off switching for the superabsorbents in (**a**) DW and (**b**) SCS. The time interval between the changes was 24 h.

Studying modulation behavior is an essential for the potential use of superabsorbents in various applications. Figure 11a,b illustrates the swelling-shrinking behavior of the prepared superabsorbent when subjected to two temperatures of 25 and 50 °C for 24 h in either DW or SCS. From the results can be found that water absorbency in both solutions at 25 °C was higher than 50 °C, whereas the p(AA-co-AM) superabsorbent did not respond to temperature stimulus. This water absorbency reduction can be attributed to destroying hydrogen bonds between hydroxyl and carboxyl groups in a superabsorbent network structure by water molecules^25^. Also, it may be that by expansion of the polymer chains in the swollen state, the water molecules can remove from the polymeric network structure easily^7^.

## Conclusion

The goal of this work was to prepare an efficient smart p(AA-co-AM-co-MA) superabsorbent with introducing maleic acid as a new structure in the synthesis of smart superabsorbents. The different analyses confirmed exactly the occurrence of the copolymerization process. Important factors in every superabsorbent are absorption aptitude, rate, strength, and retention time. In this superabsorbent, these factors are very good compared to other reported superabsorbents. The maximum water absorption capacity in DW and SCS is 1348 g/g and 106 g/g, respectively. Absorption capacity, especially in physiological solutions such as urea (1627) and D-glucose (1127) is very higher than most of the reported amount in the literature. The superabsorbent showed a good absorption rate in both DW and saline solutions. Kinetic model of water absorbency at the initial step in DW and SCS followed the mechanism of the non-Fickian type and dominant Fickian diffusion, respectively. Also, the experimental results were in good agreement with the pseudo-second-order kinetic models. The rheology analysis confirmed the preservation of the shape and strength of the superabsorbent. In regard responsibility properties of the superabsorbent, consequences showed well on–off switching reversibility behavior in salt, pH, and temperature solutions. Also, the superabsorbent was recycled and reused Five times without substantially decreasing water absorbency capacity. This superabsorbent with faster swelling, the ability to retain a large amount of water, and the ability to respond to multistimuli could be useful for a wide variety of applications, from agriculture to biomedicine fields.

## Supplementary Information

Supplementary Information.

## Data Availability

All data generated or analyzed during this study are included in this published article [its supplementary information files].
