# Supplementary material for: Maleic acid as an important monomer in synthesis of stimuli-responsive poly(acrylic acid-co-acrylamide-co-maleic acid) superabsorbent polymer
Source: Sci Rep. 2023 Mar 2;13:3511. doi: 10.1038/s41598-023-30558-3 (PMC9981600; doi:10.1038/s41598-023-30558-3)
Supplement: Supplementary file 1 — Supplementary Information. [file 41598_2023_30558_MOESM1_ESM.docx]

Supporting information

**Maleic acid as an important monomer in synthesis of stimuli-responsive poly(acrylic acid-co-acrylamide-co-maleic acid) superabsorbent** **polymer**

Fatemeh Jamali, Negar Etminani-Esfahani, Abbas Rahmati*

Department of Chemistry, University of Isfahan, P.O. Box 81746-73441, Isfahan, Iran.

^*^Corresponding author: Tel.: 98 31 37934943, E-mail address: [a.rahmati@sci.ui.ac.ir](mailto:a.rahmati@sci.ui.ac.ir)

**Data for Figure 1a**


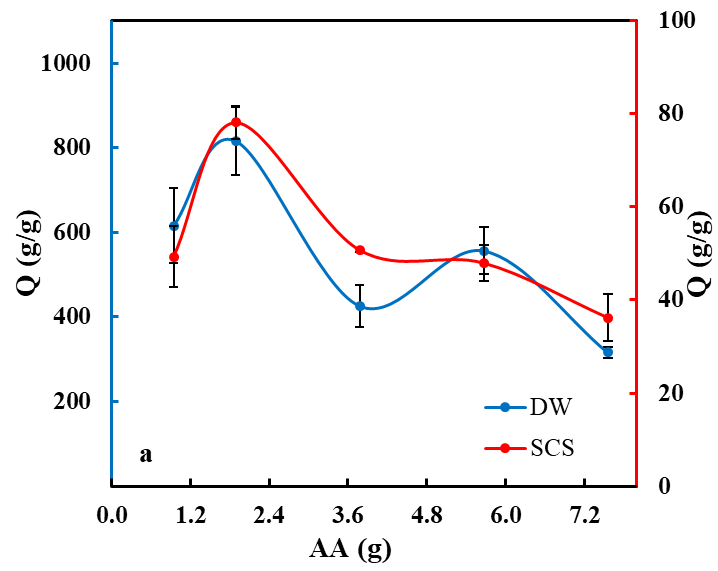


Higher absorbency:

MA: 1.47 g

AM: 1.06 g

APS: 0.018 g

NMBA: 0.0045g

NaOH: 2 g

T: 2h

| DW | | |  | |  | | |  |  | |  | | | |  | | |  | | |  | | |  | |  | |  | | |
| --- | --- | --- | --- | --- | --- | --- | --- | --- | --- | --- | --- | --- | --- | --- | --- | --- | --- | --- | --- | --- | --- | --- | --- | --- | --- | --- | --- | --- | --- | --- |
| AA (g) | AM (g) | MA (g) | | (NH_4_)_2_SO_4_ (g) | | NMBA (g) | | | | NaOH (g) | | | Time (h) | | | Q1 | | | Q2 | | | Q3 | | | Q | | SD | |  | |
| 0.95 | 1.06 | 1.47 | | 0.018 | | 0.0045 | | | | 2 | | | 2 | | | 640.64 | | | 708.84 | | | 496.92 | | | 615.50 | | 88.33 | |  | |
| 1.80 | 1.06 | 1.47 | | 0.018 | | 0.0045 | | | | 2 | | | 2 | | | 706.71 | | | 895.14 | | | 845.28 | | | 815.71 | | 79.72 | |  | |
| 3.78 | 1.06 | 1.47 | | 0.018 | | 0.0045 | | | | 2 | | | 2 | | | 450.83 | | | 470.93 | | | 357.23 | | | 426.33 | | 49.55 | |  | |
| 5.67 | 1.06 | 1.47 | | 0.018 | | 0.0045 | | | | 2 | | | 2 | | | 529.07 | | | 633.05 | | | 506.39 | | | 556.16 | | 55.14 | |  | |
| 7.56 | 1.06 | 1.47 | | 0.018 | | 0.0045 | | | | 2 | | | 2 | | | 298.62 | | | 322.02 | | | 328.35 | | | 316.33 | | 12.79 | |  | |
|  |  |  | | |  | | |  |  | | |  | | |  | | |  | | |  | | |  | |  | | |  | |
|  |  |  | | |  | | |  |  | |  | | | |  | | |  | | |  | | |  | |  | |  | | |
| SCS | |  | | |  | | |  |  | |  | | | |  | | |  | | |  | | |  | |  | |  | | |
|  |  |  | | |  | | |  |  | |  | | | |  | | |  | | |  | | |  | |  | |  | | |
| AA (g) | AM (g) | MA (g) | | (NH_4_)2SO_4_(g) | | | NMBA(g) | | | NaOH (g) | | | | Time (h) | | | Q1 | | | Q2 | | | Q3 | | Q | | SD | | |  |
| 0.95 | 1.06 | 1.47 | | 0.018 | | | 0.0045 | | | 2 | | | | 2 | | | 40.91 | | | 50.04 | | | 56.98 | | 49.31 | | 6.58 | | |  |
| 1.80 | 1.06 | 1.47 | | 0.018 | | | 0.0045 | | | 2 | | | | 2 | | | 79.45 | | | 81.77 | | | 73.20 | | 78.14 | | 3.62 | | |  |
| 3.78 | 1.06 | 1.47 | | 0.018 | | | 0.0045 | | | 2 | | | | 2 | | | 50.29 | | | 51.15 | | | 50.84 | | 50.76 | | 0.36 | | |  |
| 5.67 | 1.06 | 1.47 | | 0.018 | | | 0.0045 | | | 2 | | | | 2 | | | 47.46 | | | 52.93 | | | 43.49 | | 47.96 | | 3.87 | | |  |
| 7.56 | 1.06 | 1.47 | | 0.018 | | | 0.0045 | | | 2 | | | | 2 | | | 34.68 | | | 43.02 | | | 30.93 | | 36.21 | | 5.05 | | |  |

**Data for Figure 1b**


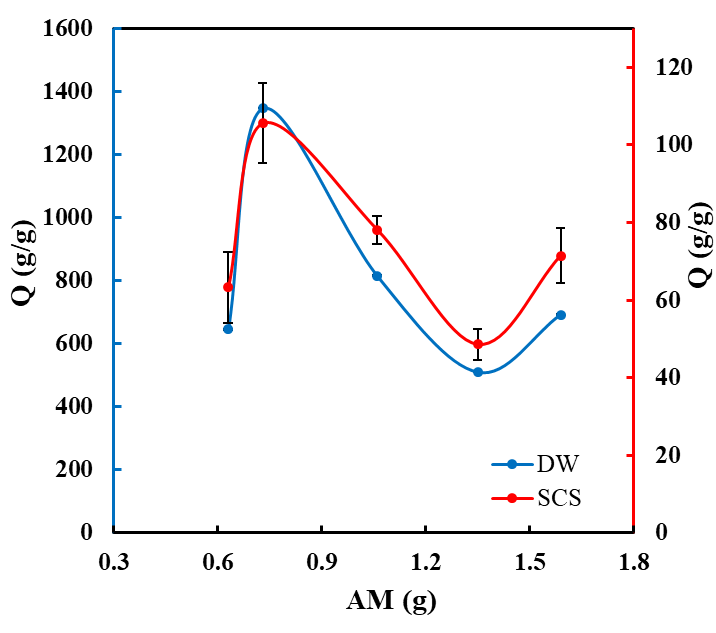


Higher absorbency:

MA: 1.47 g

AA: 1.89 g

APS: 0.018 g

NMBA: 0.0045 g

NaOH: 2 g

T: 2h

**b**

| DW | |  |  |  |  |  |  |  |  |  |  |
| --- | --- | --- | --- | --- | --- | --- | --- | --- | --- | --- | --- |
| AA (g) | AM (g) | MA (g) | (NH_4_)_2_SO_4_ (g) | NMBA (g) | NaOH (g) | Time (h) | Q1 | Q2 | Q3 | Q | SD |
| 1.8 | 0.63 | 1.47 | 0.018 | 0.0045 | 2 | 2 | 856.45 | 556.00 | 525.29 | 645.91 | 149.40 |
| 1.8 | 0.73 | 1.47 | 0.018 | 0.0045 | 2 | 2 | 1458.99 | 1131.24 | 1452.93 | 1347.72 | 153.09 |
| 1.8 | 1.06 | 1.47 | 0.018 | 0.0045 | 2 | 2 | 706.71 | 895.14 | 845.283 | 815.71 | 79.72 |
| 1.8 | 1.35 | 1.47 | 0.018 | 0.0045 | 2 | 2 | 508.62 | 514.20 | 507.18 | 510.00 | 3.03 |
| 1.8 | 1.59 | 1.47 | 0.018 | 0.0045 | 2 | 2 | 701.48 | 560.56 | 815.21 | 692.44 | 104.16 |
|  |  |  |  |  |  |  |  |  |  |  |  |
|  |  |  |  |  |  |  |  |  |  |  |  |
| SCS | |  |  |  |  |  |  |  |  |  |  |
|  |  |  |  |  |  |  |  |  |  |  |  |
| AA (g) | AM (g) | MA (g) | (NH_4_)_2_SO_4_ (g) | NMBA (g) | NaOH (g) | Time (h) | Q1 | Q2 | Q3 | Q | SD |
| 1.8 | 0.63 | 1.47 | 0.018 | 0.0045 | 2 | 2 | 60.03 | 75.57 | 54.09 | 63.23 | 9.06 |
| 1.8 | 0.73 | 1.47 | 0.018 | 0.0045 | 2 | 2 | 98.52 | 120.3 | 98.05 | 105.62 | 10.38 |
| 1.8 | 1.06 | 1.47 | 0.018 | 0.0045 | 2 | 2 | 79.45 | 81.77 | 73.20 | 78.14 | 3.62 |
| 1.8 | 1.35 | 1.47 | 0.018 | 0.0045 | 2 | 2 | 43.57 | 53.24 | 48.90 | 48.57 | 3.95 |
| 1.8 | 1.59 | 1.47 | 0.018 | 0.0045 | 2 | 2 | 71.37 | 80.11 | 62.67 | 71.45 | 7.12 |

**Data for Figure 1c**


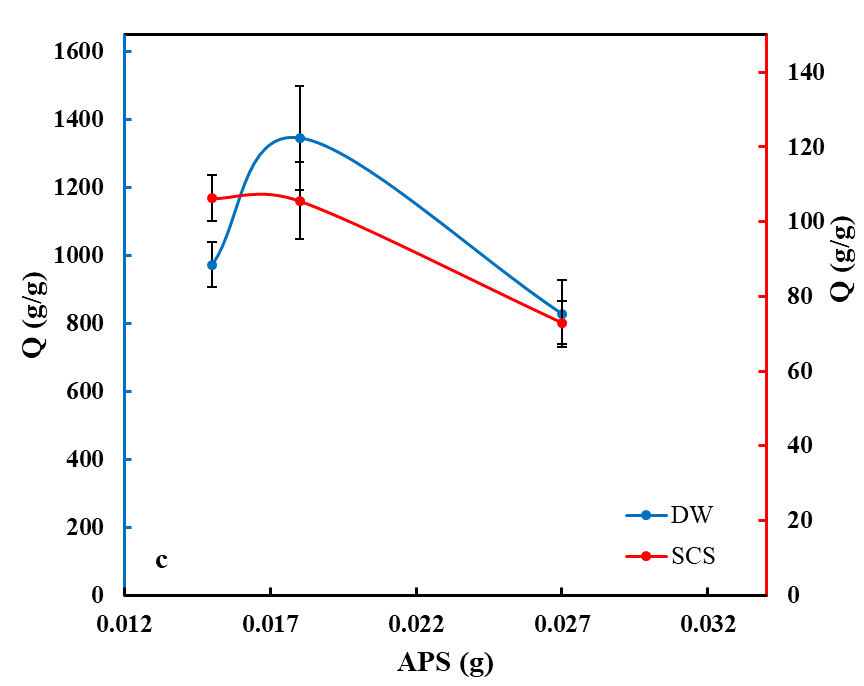


Higher absorbency:

MA: 1.47 g

AA: 1.89 g

AM: 0.73 g

NMBA: 0.0045 g

NaOH: 2 g

T: 2h

| DW | |  |  |  |  |  |  |  |  |  |  |
| --- | --- | --- | --- | --- | --- | --- | --- | --- | --- | --- | --- |
| AA (g) | AM (g) | MA (g) | (NH_4_)_2_SO_4_ (g) | NMBA (g) | NaOH (g) | Time (h) | Q1 | Q2 | Q3 | Q | SD |
| 1.8 | 0.73 | 1.47 | 0.015 | 0.0045 | 2 | 2 | 1016.60 | 1024.91 | 879.82 | 973.78 | 66.52 |
| 1.8 | 0.73 | 1.47 | 0.018 | 0.0045 | 2 | 2 | 1458.99 | 1131.24 | 1452.93 | 1347.72 | 153.09 |
| 1.8 | 0.73 | 1.47 | 0.027 | 0.0045 | 2 | 2 | 910.40 | 885.86 | 689.11 | 828.46 | 99.04 |
|  |  |  |  |  |  |  |  |  |  |  |  |
|  |  |  |  |  |  |  |  |  |  |  |  |
|  |  |  |  |  |  |  |  |  |  |  |  |
| SCS | |  |  |  |  |  |  |  |  |  |  |
| AA (g) | AM (g) | MA (g) | (NH_4_)_2_SO_4_ (g) | NMBA (g) | NaOH (g) | Time (h) | Q1 | Q2 | Q3 | Q | SD |
| 1.8 | 0.73 | 1.47 | 0.015 | 0.0045 | 2 | 2 | 106.01 | 117.87 | 95.09 | 106.31 | 6.09 |
| 1.8 | 0.73 | 1.47 | 0.018 | 0.0045 | 2 | 2 | 98.52 | 120.30 | 98.05 | 105.62 | 10.38 |
| 1.8 | 0.73 | 1.47 | 0.027 | 0.0045 | 2 | 2 | 64.98 | 78.86 | 75.01 | 72.95 | 5.85 |

**Data for Figure 1d**


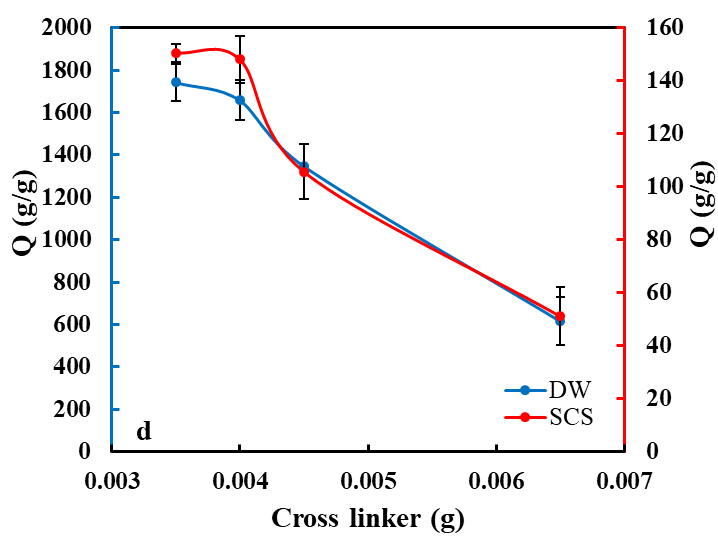


Higher absorbency:

MA: 1.47 g

AA: 1.89 g

AM: 0.73 g

APS: 0.018 g

NaOH: 2 g

T: 2 h

| DW | |  |  |  |  |  |  |  |  |  |  |
| --- | --- | --- | --- | --- | --- | --- | --- | --- | --- | --- | --- |
| AA (g) | AM (g) | MA (g) | (NH_4_)_2_SO_4_ (g) | NMBA (g) | NaOH (g) | Time (h) | Q1 | Q2 | Q3 | Q | SD |
| 1.89 | 0.73 | 1.47 | 0.018 | 0.0035 | 2 | 2 | 1629.25 | 1762 | 1839.53 | 1743.59 | 86.83 |
| 1.89 | 0.73 | 1.47 | 0.018 | 0.0040 | 2 | 2 | 1585.40 | 1791.10 | 1601.70 | 1659.40 | 93.36 |
| 1.89 | 0.73 | 1.47 | 0.018 | 0.0045 | 2 | 2 | 1458.99 | 1131.24 | 1452.93 | 1347.72 | 153.09 |
| 1.89 | 0.73 | 1.47 | 0.018 | 0.0065 | 2 | 2 | 741.19 | 466.87 | 638.50 | 615.52 | 113.16 |
|  |  |  |  |  |  |  |  |  |  |  |  |
| SCS | |  |  |  |  |  |  |  |  |  |  |
| AA (g) | AM (g) | MA (g) | (NH_4_)_2_SO_4_ (g) | NMBA (g) | NaOH (g) | Time (h) | Q1 | Q2 | Q3 | Q | SD |
| 1.8 | 0.73 | 1.47 | 0.018 | 0.0035 | 2 | 2 | 145.91 | 154.33 | 151.14 | 150.46 | 3.47 |
| 1.8 | 0.73 | 1.47 | 0.018 | 0.0040 | 2 | 2 | 135.61 | 152.02 | 156.46 | 148.03 | 8.97 |
| 1.8 | 0.73 | 1.47 | 0.018 | 0.0045 | 2 | 2 | 98.52 | 120.30 | 98.05 | 105.62 | 10.38 |
| 1.8 | 0.73 | 1.47 | 0.018 | 0.0065 | 2 | 2 | 38.75 | 49.08 | 65.35 | 51.06 | 10.95 |

**Data for Figure 1e**


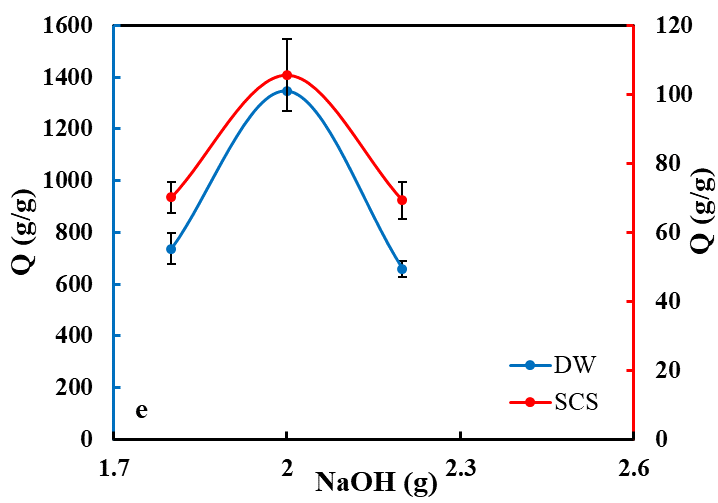


Higher absorbency:

MA: 1.47 g

AA: 1.89 g

AM: 0.73 g

APS: 0.018 g

NMBA: 0.0045 g

T: 2 h

| DW | |  |  |  |  |  |  |  |  |  |  |
| --- | --- | --- | --- | --- | --- | --- | --- | --- | --- | --- | --- |
| AA (g) | AM (g) | MA (g) | (NH_4_)_2_SO_4_ (g) | NMBA (g) | NaOH (g) | Time (h) | Q1 | Q2 | Q3 | Q | SD |
| 1.8 | 0.73 | 1.47 | 0.018 | 0.0045 | 1.8 | 2 | 727.45 | 670.10 | 813.17 | 736.91 | 58.79 |
| 1.8 | 0.73 | 1.47 | 0.018 | 0.0045 | 2.0 | 2 | 1458.99 | 1131.24 | 1452.93 | 1347.72 | 153.09 |
| 1.8 | 0.73 | 1.47 | 0.018 | 0.0045 | 2.2 | 2 | 650.30 | 701.20 | 625.80 | 659.10 | 31.40 |
|  |  |  |  |  |  |  |  |  |  |  |  |
|  |  |  |  |  |  |  |  |  |  |  |  |
| SCS | |  |  |  |  |  |  |  |  |  |  |
| AA (g) | AM (g) | MA (g) | (NH_4_)_2_SO_4_ (g) | NMBA (g) | NaOH (g) | Time (h) | Q1 | Q2 | Q3 | Q | SD |
| 1.8 | 0.73 | 1.47 | 0.018 | 0.0045 | 1.8 | 2 | 64.21 | 75.04 | 71.38 | 70.21 | 4.50 |
| 1.8 | 0.73 | 1.47 | 0.018 | 0.0045 | 2.0 | 2 | 98.52 | 120.30 | 98.05 | 105.62 | 10.38 |
| 1.8 | 0.73 | 1.47 | 0.018 | 0.0045 | 2.2 | 2 | 63.25 | 68.30 | 76.26 | 69.27 | 5.35 |

**Data for Figure 1f**


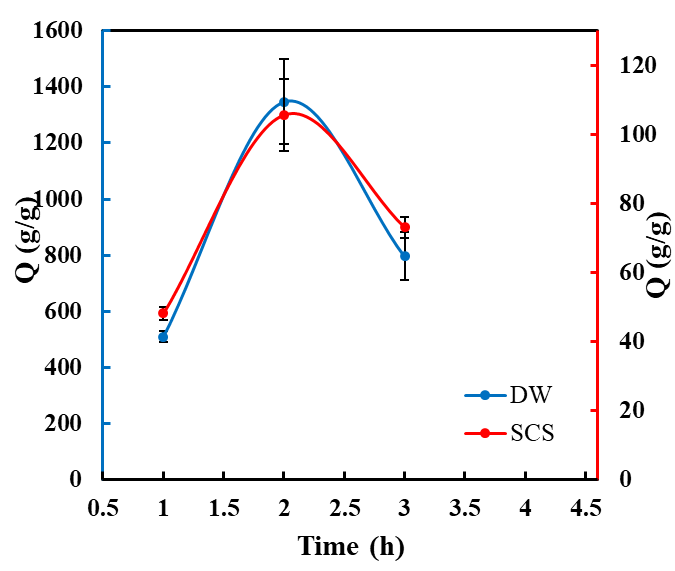


Higher absorbency:

MA: 1.47 g

AA: 1.89 g

AM: 0.73 g

APS: 0.018 g

NMBA: 0.0045 g

NaOH: 2 g

**f**

| DW | | |  | |  | |  |  |  |  |  |  |  |  |
| --- | --- | --- | --- | --- | --- | --- | --- | --- | --- | --- | --- | --- | --- | --- |
| AA (g) | AM (g) | MA (g) | | (NH_4_)_2_SO_4_ (g) | | NMBA (g) | | NaOH (g) | Time (h) | Q1 | Q2 | Q3 | Q | SD |
| 1.8 | 0.73 | 1.47 | | 0.018 | | 0.0045 | | 2.0 | 1 | 530.08 | 512.87 | 485.42 | 509.45 | 18.39 |
| 1.8 | 0.73 | 1.47 | | 0.018 | | 0.0045 | | 2.0 | 2 | 1458.99 | 1131.24 | 1452.93 | 1347.72 | 153.09 |
| 1.8 | 0.73 | 1.47 | | 0.018 | | 0.0045 | | 2.0 | 3 | 674.66 | 864.34 | 847.34 | 795.42 | 85.69 |
|  |  |  | |  | |  | |  |  |  |  |  |  |  |
|  |  |  | |  | |  | |  |  |  |  |  |  |  |
|  |  |  | |  | |  | |  |  |  |  |  |  |  |
|  |  |  | |  | |  | |  |  |  |  |  |  |  |
| SCS | |  | |  | |  | |  |  |  |  |  |  |  |
| AA (g) | AM (g) | MA (g) | | (NH_4_)_2_SO_4_ (g) | | NMBA (g) | | NaOH (g) | Time (h) | Q1 | Q2 | Q3 | Q | SD |
| 1.8 | 0.73 | 1.47 | | 0.018 | | 0.0045 | | 2.0 | 1 | 47.24 | 50.69 | 46.34 | 48.09 | 1.87 |
| 1.8 | 0.73 | 1.47 | | 0.018 | | 0.0045 | | 2.0 | 2 | 98.52 | 120.30 | 98.05 | 105.62 | 10.38 |
| 1.8 | 0.73 | 1.47 | | 0.018 | | 0.0045 | | 2.0 | 3 | 69.12 | 71.13 | 75.88 | 73.10 | 3.02 |

**FT-IR spectrum p(AA-co-AM-co-MA)**


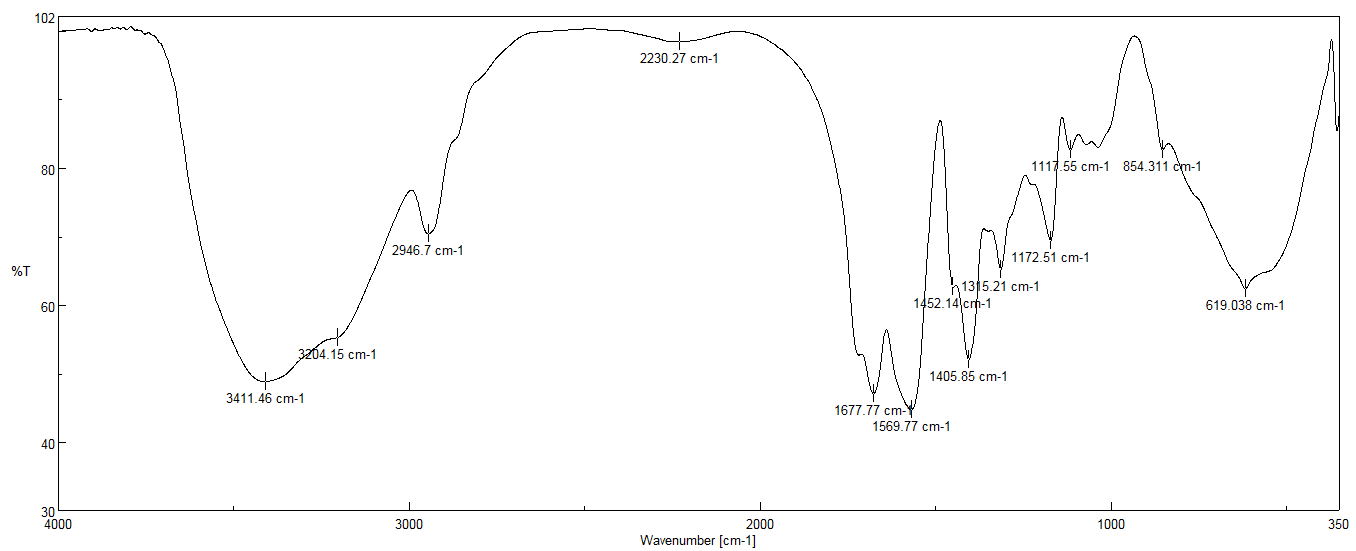


**FT-IR spectrum p(AA-co-AM)**


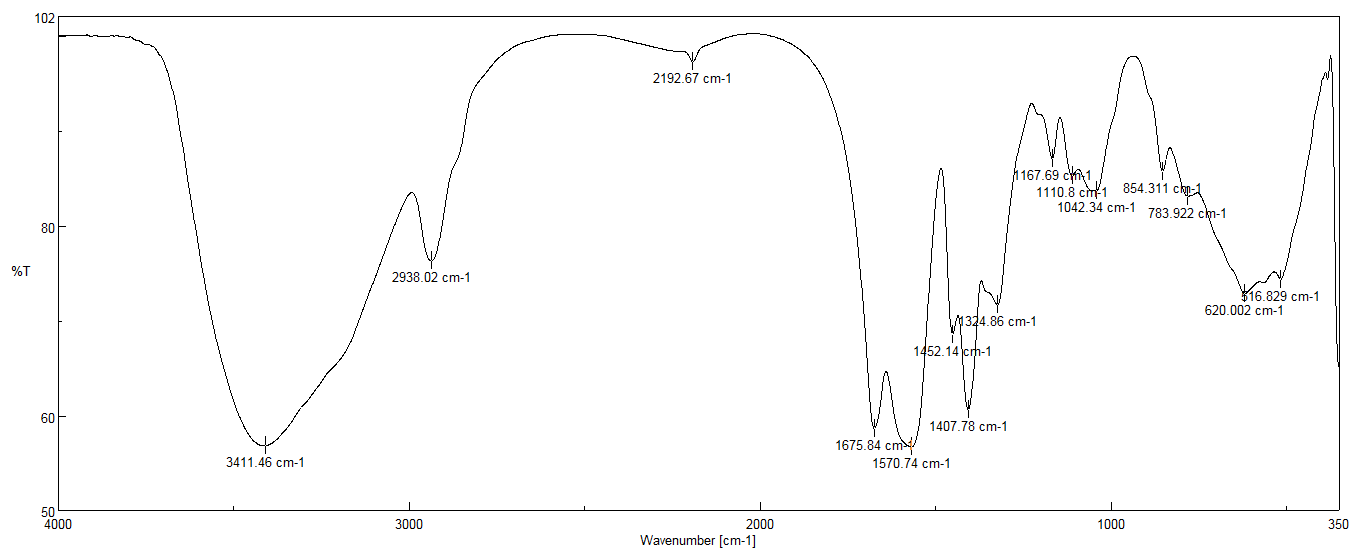


**TGA and DSC p(AA-co-AM-co-MA)**


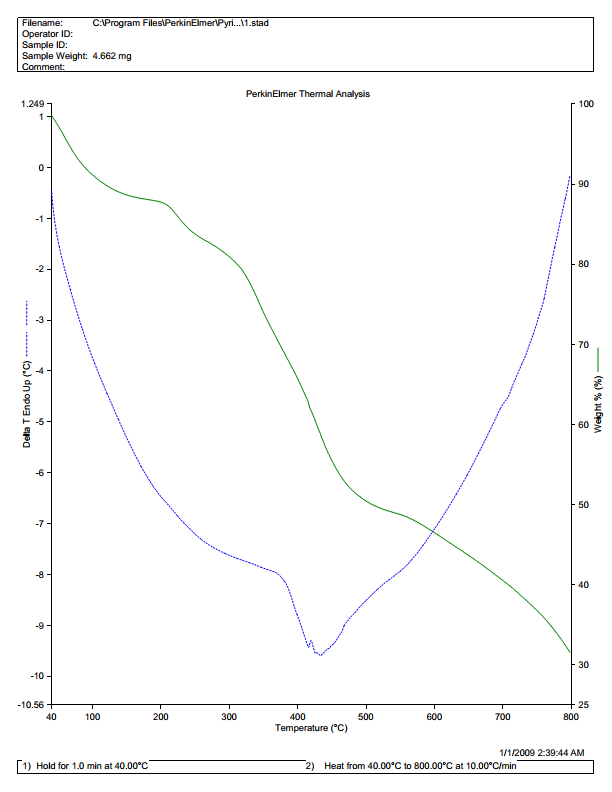


**TGA and DTG of p(AA-co-AM)**

**
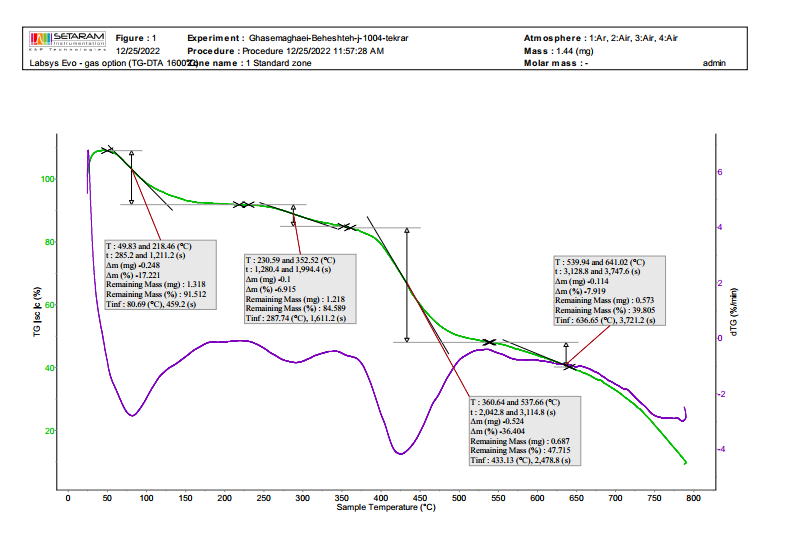
**

**SEM micrographs of p(AA-co-AM-co-MA) superabsorbent (c, d),**








**
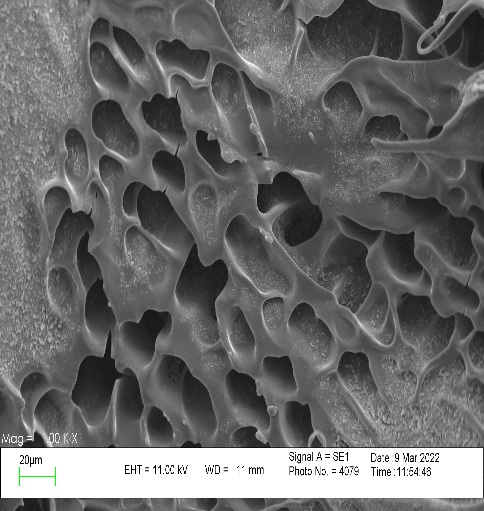
SEM micrographs of p(AA-co-AM) superabsorbent (e, f).**

**
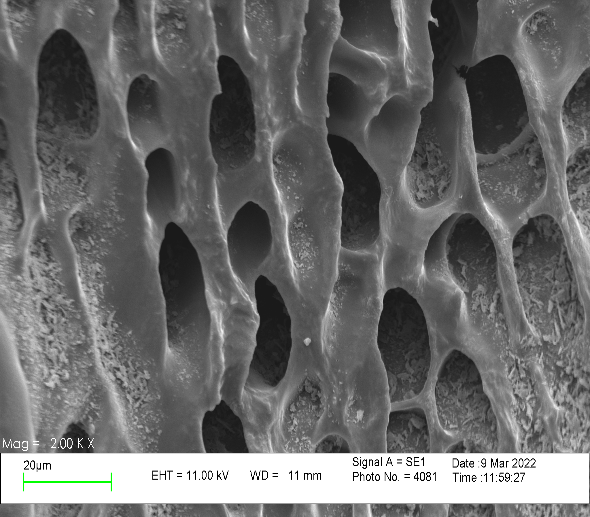
**

**Rheological analysis (Figure 4)**

**G' and G" as a function of shear stress for superabsorbent with water absorbency 1348 g/g in fig 4a**

**G' and G" as a function of shear stress for superabsorbent with water absorbency 1744 g/g in fig 4a**

**tan δ versus shear stress for superabsorbent with water absorbency 1348 g/g in fig 4b**

**tan δ versus shear stress for superabsorbent with water absorbency 1744 g/g in fig 4b**

**Phase angle versus shear stress for superabsorbent with water absorbency 1348 g/g in fig 4c**

**Phase angle versus shear stress for superabsorbent with water absorbency 1744 g/g in fig 4c**

**G' and G" versus angular frequency for superabsorbent with water absorbency 1348 g/g in fig 4d**

**G' and G" versus angular frequency for superabsorbent with water absorbency 1744 g/g in fig 4d**

**Data for Figure 5a**

| DW |  |  |  |  |  |  |
| --- | --- | --- | --- | --- | --- | --- |
| Time |  | Q1 | Q2 | Q3 | Q | SD |
| 0 |  | 0 | 0 | 0 | 0 | 0 |
| 60 |  | 394.36 | 361.81 | 398.15 | 384.70 | 16.31 |
| 120 |  | 894.10 | 812.71 | 782.73 | 829.85 | 47.05 |
| 180 |  | 948.15 | 913.95 | 1013.45 | 958.50 | 41.28 |
| 240 |  | 1216.09 | 1030.09 | 1205.59 | 1150.59 | 85.31 |
| 300 |  | 1397.91 | 1255.29 | 1202.43 | 1285.21 | 82.56 |
| 360 |  | 1338.35 | 1250.84 | 1449.61 | 1346.26 | 81.34 |
| 420 |  | 1320.26 | 1350.12 | 1418.92 | 1363.00 | 41.31 |
| 480 |  | 1320.26 | 1350.12 | 1418.92 | 1363.00 | 41.31 |
| 540 |  | 1320.26 | 1350.12 | 1418.92 | 1363.00 | 41.31 |
| 600 |  | 1320.26 | 1350.12 | 1418.92 | 1363.00 | 41.31 |
| 660 |  | 1320.26 | 1350.12 | 1418.92 | 1363.00 | 41.31 |
| 720 |  | 1320.26 | 1350.12 | 1418.92 | 1363.00 | 41.31 |
| 780 |  | 1320.26 | 1350.12 | 1418.92 | 1363.00 | 41.31 |
| 840 |  | 1320.26 | 1350.12 | 1418.92 | 1363.00 | 41.31 |
| 900 |  | 1320.26 | 1350.12 | 1418.92 | 1363.00 | 41.31 |
| 960 |  | 1320.26 | 1350.12 | 1418.92 | 1363.00 | 41.31 |

**Data for Figure 5b**

| SCS | |  |  |  |  |  |
| --- | --- | --- | --- | --- | --- | --- |
| Time |  | Q1 | Q2 | Q3 | Q | SD |
| 0 |  | 0 | 0 | 0 | 0 | 0 |
| 60 |  | 57.20 | 44.81 | 58.65 | 53.55 | 6.21 |
| 120 |  | 71.50 | 71.81 | 76.25 | 73.20 | 2.17 |
| 180 |  | 81.50 | 89.9 | 81.10 | 84.20 | 4.06 |
| 240 |  | 90.04 | 84.93 | 102.36 | 92.40 | 7.32 |
| 300 |  | 101.38 | 89.75 | 113.02 | 101.38 | 9.50 |
| 360 |  | 101.38 | 89.75 | 113.02 | 101.38 | 9.50 |
| 420 |  | 101.38 | 89.75 | 113.02 | 101.38 | 9.50 |
| 480 |  | 101.38 | 89.75 | 113.02 | 101.38 | 9.50 |
| 540 |  | 101.38 | 89.75 | 113.02 | 101.38 | 9.50 |
| 600 |  | 101.38 | 89.75 | 113.02 | 101.38 | 9.50 |
| 660 |  | 101.38 | 89.75 | 113.02 | 101.38 | 9.50 |
| 720 |  | 101.38 | 89.75 | 113.02 | 101.38 | 9.50 |
| 780 |  | 101.38 | 89.75 | 113.02 | 101.38 | 9.50 |
| 840 |  | 101.38 | 89.75 | 113.02 | 101.38 | 9.50 |
| 900 |  | 101.38 | 89.75 | 113.02 | 101.38 | 9.50 |
| 960 |  | 101.38 | 89.75 | 113.02 | 101.38 | 9.50 |

**Data for Figure 5c**

**c**

| DW |  |  |  | |  | |  |  | SCS |  |  |  |  |
| --- | --- | --- | --- | --- | --- | --- | --- | --- | --- | --- | --- | --- | --- |
| Time | LN(T) | Q | | F | | LN(F) |  |  | Time | LN(T) | Q | F | LN(F) |
| 0 | #NUM! | 0 | | 0 | | #NUM! |  |  | 0 | #NUM! | 0 | 0 | #NUM! |
| 60 | 4.09 | 384.70 | | 0.28 | | -1.26 |  |  | 60 | 4.09 | 53.55 | 0.53 | -0.64 |
| 120 | 4.79 | 829.80 | | 0.61 | | -0.50 |  |  | 120 | 4.79 | 73.20 | 0.72 | -0.33 |
| 180 | 5.19 | 958.50 | | 0.70 | | -0.35 |  |  | 180 | 5.19 | 84.20 | 0.83 | -0.19 |
| 240 | 5.48 | 1150.59 | | 0.84 | | -0.17 |  |  | 240 | 5.48 | 92.40 | 0.91 | -0.09 |
| 300 | 5.70 | 1285.21 | | 0.94 | | -0.06 |  |  | 300 | 5.70 | 101.38 | 1.00 | 0.00 |
| 360 | 5.89 | 1346.26 | | 0.99 | | -0.01 |  |  | 360 | 5.89 | 101.38 | 1.00 | 0.00 |
| 420 | 6.04 | 1363 | | 1.00 | | 0.00 |  |  | 420 | 6.04 | 101.38 | 1.00 | 0.00 |
| 480 | 6.17 | 1363 | | 1.00 | | 0.00 |  |  | 480 | 6.17 | 101.38 | 1.00 | 0.00 |
| 540 | 6.29 | 1363 | | 1.00 | | 0.00 |  |  | 540 | 6.29 | 101.38 | 1.00 | 0.00 |
| 600 | 6.40 | 1363 | | 1.00 | | 0.00 |  |  | 600 | 6.40 | 101.38 | 1.00 | 0.00 |
| 660 | 6.49 | 1363 | | 1.00 | | 0.00 |  |  | 660 | 6.49 | 101.38 | 1.00 | 0.00 |
| 720 | 6.58 | 1363 | | 1.00 | | 0.00 |  |  | 720 | 6.58 | 101.38 | 1.00 | 0.00 |
| 780 | 6.66 | 1363 | | 1.00 | | 0.00 |  |  | 780 | 6.66 | 101.38 | 1.00 | 0.00 |
| 840 | 6.73 | 1363 | | 1.00 | | 0.00 |  |  | 840 | 6.73 | 101.38 | 1.00 | 0.00 |
| 900 | 6.80 | 1363 | | 1.00 | | 0.00 |  |  | 900 | 6.80 | 101.38 | 1.00 | 0.00 |
| 960 | 6.87 | 1363 | | 1.00 | | 0.00 |  |  | 960 | 6.87 | 101.38 | 1.00 | 0.00 |

**Data for Figure 5d**

| DW |  | |  | |  | |  | SCS |  |  |
| --- | --- | --- | --- | --- | --- | --- | --- | --- | --- | --- |
| Time | | Q | | t/Qt | |  |  | Time | Q | t/Qt |
| 0 | | 0 | | #DIV/0! | |  |  | 0 | 0 | #DIV/0! |
| 60 | | 0 | | #DIV/0! | |  |  | 60 | 53.55 | 1.12 |
| 120 | | 829.80 | | 0.14 | |  |  | 120 | 73.20 | 1.64 |
| 180 | | 958.50 | | 0.19 | |  |  | 180 | 84.20 | 2.14 |
| 240 | | 1150.59 | | 0.21 | |  |  | 240 | 92.40 | 2.60 |
| 300 | | 1285.21 | | 0.23 | |  |  | 300 | 101.38 | 2.60 |
| 360 | | 1346.26 | | 0.27 | |  |  | 360 | 101.38 | 3.55 |
| 420 | | 1363.00 | | 0.31 | |  |  | 420 | 101.38 | 4.14 |
| 480 | | 1363.00 | | 0.35 | |  |  | 480 | 101.38 | 4.73 |
| 540 | | 1363.00 | | 0.40 | |  |  | 540 | 101.38 | 5.33 |
| 600 | | 1363.00 | | 0.44 | |  |  | 600 | 101.38 | 5.92 |
| 660 | | 1363.00 | | 0.48 | |  |  | 660 | 101.38 | 6.51 |
| 720 | | 1363.00 | | 0.53 | |  |  | 720 | 101.38 | 7.10 |
| 780 | | 1363.00 | | 0.57 | |  |  | 780 | 101.38 | 7.69 |
| 840 | | 1363.00 | | 0.62 | |  |  | 840 | 101.38 | 8.29 |
| 900 | | 1363.00 | | 0.66 | |  |  | 900 | 101.38 | 8.88 |
| 960 | | 1363.00 | | 0.70 | |  |  | 960 | 101.38 | 9.47 |

**Data for Figure 6a**

| DW |  |  |  |  |  |  |
| --- | --- | --- | --- | --- | --- | --- |
| 25 |  | Q1 | Q2 | Q3 | Q | SD |
| time |  |  |  |  |  |  |
| 60 |  | 1459.85 | 1564.15 | 1486.40 | 1503.00 | 44.26 |
| 120 |  | 1283.50 | 1433.15 | 1413.48 | 1376.71 | 66.40 |
| 180 |  | 1186.15 | 1370.85 | 1320.80 | 1292.60 | 77.99 |
| 240 |  | 1092.90 | 1292.12 | 1220.68 | 1201.93 | 82.41 |
| 300 |  | 1039.05 | 1235.00 | 1199.32 | 1157.79 | 85.22 |
| 360 |  | 1189.45 | 1000.15 | 1043.52 | 1077.57 | 80.97 |
| 420 |  | 884.40 | 1098.05 | 1021.96 | 1001.47 | 88.42 |
| 480 |  | 833.75 | 1057.20 | 974.24 | 955.06 | 92.23 |

| SCS |  |  |  |  |  |  |
| --- | --- | --- | --- | --- | --- | --- |
| 25^o^C |  | Q1 | Q2 | Q3 | Q | SD |
| time |  |  |  |  |  |  |
| 60 |  | 102.57 | 102.72 | 107.44 | 104.24 | 2.26 |
| 120 |  | 75.25 | 73.05 | 67.31 | 71.78 | 3.35 |
| 180 |  | 45.36 | 49.40 | 58.81 | 51.19 | 5.63 |
| 240 |  | 41.60 | 35.86 | 28.77 | 35.41 | 5.25 |
| 300 |  | 25.75 | 19.72 | 17.05 | 20.84 | 3.64 |
| 360 |  | 23.18 | 11.28 | 9.56 | 14.70 | 6.06 |
| 420 |  | 19.38 | 10.65 | 7.43 | 12.49 | 5.05 |
| 480 |  | 19.38 | 10.65 | 7.43 | 12.49 | 5.05 |

**Data for Figure 6b**

| DW |  |  |  |  |  |  |
| --- | --- | --- | --- | --- | --- | --- |
| 50 ^o^C |  |  |  |  |  |  |
| time |  | Q1 | Q2 | Q3 | Q |  |
| 60 |  | 1149.57 | 1107.64 | 965.15 | 1074.12 | 78.93 |
| 120 |  | 880.81 | 668.40 | 697.60 | 748.94 | 94.01 |
| 180 |  | 548.12 | 527.70 | 404.40 | 493.40 | 63.49 |
| 240 |  | 492.86 | 388.45 | 257.40 | 379.57 | 96.33 |
| 300 |  | 386.70 | 296.68 | 184.30 | 289.23 | 82.80 |
| 360 |  | 233.52 | 165.91 | 121.80 | 173.74 | 45.94 |
| 420 |  | 146.43 | 87.54 | 67.70 | 100.56 | 33.43 |
| 480 |  | 146.43 | 87.54 | 67.70 | 100.56 | 33.43 |

| SCS |  |  |  |  |  |  |
| --- | --- | --- | --- | --- | --- | --- |
| 50 oC |  |  |  |  |  |  |
| time |  | Q1 | Q2 | Q3 | Q | SD |
| 60 |  | 103.43 | 106.41 | 84.40 | 98.08 | 9.75 |
| 120 |  | 36.24 | 50.00 | 43.32 | 43.20 | 5.62 |
| 180 |  | 15.33 | 24.33 | 12.04 | 17.20 | 5.19 |
| 240 |  | 3.50 | 6.10 | 3.50 | 4.40 | 1.23 |
| 300 |  | 1.00 | 1.20 | 1.04 | 1.10 | 0.09 |
| 360 |  | 0.90 | 1.10 | 0.86 | 0.95 | 0.11 |
| 420 |  | 0.90 | 1.10 | 0.86 | 0.95 | 0.11 |
| 480 |  | 0.90 | 1.10 | 0.86 | 0.95 | 0.11 |

**Data for Figure 7a**

| DW |  | |  |  | |  | |  | |  | |  |
| --- | --- | --- | --- | --- | --- | --- | --- | --- | --- | --- | --- | --- |
| n |  | Q1 | | | Q2 | | Q3 | | Q | | SD | |
| 1 |  | 1458.99 | | | 1131.24 | | 1452.93 | | 1347.72 | | 153.09 | |
| 2 |  | 1218.10 | | | 1320.54 | | 1179.10 | | 1239.25 | | 59.65 | |
| 3 |  | 1131.10 | | | 1009.40 | | 1100.63 | | 1080.40 | | 51.71 | |
| 4 |  | 918.80 | | | 973.25 | | 953.42 | | 936.82 | | 25.35 | |
| 5 |  | 935.42 | | | 920.42 | | 915.50 | | 923.90 | | 8.47 | |

**Data for Figure 7b**

| SCS |  | |  | |  |  |  | |  | |  |
| --- | --- | --- | --- | --- | --- | --- | --- | --- | --- | --- | --- |
| n |  | Q1 | | Q2 | | Q3 | | Q | | SD | |
| 1 |  | 98.52 | | 120.30 | | 98.05 | | 105.62 | | 10.38 | |
| 2 |  | 97.34 | | 94.92 | | 112.72 | | 101.70 | | 7.88 | |
| 3 |  | 89.03 | | 90.11 | | 85.61 | | 87.25 | | 2.16 | |
| 4 |  | 74.26 | | 87.69 | | 81.92 | | 81.30 | | 5.50 | |
| 5 |  | 47.53 | | 44.60 | | 49.00 | | 47.04 | | 1.83 | |

**Data for Figure 8**

|  |  |  | Q1 | Q2 | Q3 | Q | SD |
| --- | --- | --- | --- | --- | --- | --- | --- |
| Distilled water | |  |  |  |  | 1347.92 | 153.09 |
| D-glucose solution | |  | 1113.00 | 1127.38 | 1141.76 | 1127.38 | 11.74 |
| urea solution | |  | 1550.39 | 1862.12 | 1469.25 | 1627.25 | 169.35 |
| physiological saline water | | | 91.70 | 88.20 | 95.82 | 91.91 | 3.11 |
| synthetic urine | |  | 77.60 | 69.40 | 64.50 | 70.50 | 5.40 |
| 1 wt.% NaCl solution | | |  |  |  | 105.62 | 10.37 |

**Data for Figure 9a**

| NaCl | Q1 | Q2 | Q3 | Q | SD | Q (DW) | f | SD(F) |
| --- | --- | --- | --- | --- | --- | --- | --- | --- |
| 0.2 | 135.40 | 181.91 | 175.95 | 164.45 | 18.53 | 1347.72 | 0.88 | 0.02 |
| 1 | 98.52 | 120.30 | 98.05 | 105.62 | 10.37 | 1347.72 | 0.93 | 0.02 |
| MgCl2 |  |  |  |  |  |  |  | 0.00 |
| 0.2 | 88.08 | 75.62 | 97.45 | 87.05 | 8.95 | 1347.72 | 0.94 | 0.01 |
| 1 | 39.00 | 47.22 | 53.05 | 46.42 | 5.75 | 1347.72 | 0.97 | 0.00 |
| FeCl3 |  |  |  |  |  |  |  | 0.00 |
| 0.2 | 24.08 | 27.83 | 37.31 | 29.74 | 5.58 | 1347.72 | 0.98 | 0.01 |
| 1 | 14.05 | 16.24 | 23.51 | 17.93 | 4.08 | 1347.72 | 0.99 | 0.01 |

**Data for Figure 9b**

**Data for p(AA-co-AM-co-MA)**

| Time | Q1 | | Q2 | | Q3 | | Q | | SD | |
| --- | --- | --- | --- | --- | --- | --- | --- | --- | --- | --- |
| 20 | 88.69 | | 71.79 | | 104.25 | | 88.27 | | 13.26 | |
| 40 | 139.05 | | 193.25 | | 198.97 | | 177.09 | | 26.99 | |
| 60 | 220.62 | | 232.25 | | 298.74 | | 250.50 | | 34.41 | |
| 80 | 278.60 | | 309.42 | | 331.90 | | 306.40 | | 21.85 | |
| 100 | 213.50 | | 221.20 | | 238.10 | | 224.30 | | 10.27 | |
| 120 | 155.12 | | 171.13 | | 154.72 | | 160.32 | | 7.64 | |
| 140 | 140.23 | | 134.00 | | 196.03 | | 137.11 | | 14.38 | |
| 180 | 154.06 | | 162.13 | | 159.31 | | 154.06 | | 5.56 | |
| 200 | 191.05 | | 176.92 | | 223.13 | | 197.03 | | 19.33 | |
| 220 | 151.12 | | 155.22 | | 146.06 | | 150.80 | | 3.75 | |
| 240 | 123.09 | | 125.10 | | 136.17 | | 128.12 | | 5.75 | |
| 260 | 115.50 | | 127.08 | | 72.01 | | 104.88 | | 23.71 | |
| 280 | 132.10 | | 128.50 | | 121.03 | | 127.21 | | 4.61 | |
| 300 | 137.19 | | 147.12 | | 139.74 | | 141.35 | | 4.21 | |
| 320 | 184.13 | | 178.61 | | 206.87 | | 189.87 | | 12.23 | |
| 340 | 118.27 | | 111.92 | | 140.10 | | 126.01 | | 12.34 | |
| 360 | 113.25 | | 93.33 | | 129.55 | | 112.04 | | 14.81 | |
| 380 | 86.01 | | 106.01 | | 129.32 | | 107.11 | | 17.70 | |
|  |  |  | |  | |  | |  | |  |

**Data for p(AA-co-AM)**

| Time | Q1 | | Q2 | | Q3 | | Q | | SD | |
| --- | --- | --- | --- | --- | --- | --- | --- | --- | --- | --- |
| 20 | 73.05 | | 69.03 | | 74.37 | | 72.15 | | 2.78 | |
| 40 | 138.51 | | 142.61 | | 119.32 | | 133.48 | | 12.43 | |
| 60 | 160.55 | | 167.01 | | 166.69 | | 164.75 | | 3.64 | |
| 80 | 176.42 | | 182.64 | | 198.64 | | 185.90 | | 11.46 | |
| 100 | 139.02 | | 155.12 | | 150.73 | | 148.29 | | 8.32 | |
| 120 | 119.19 | | 125.21 | | 123.91 | | 122.77 | | 3.17 | |
| 140 | 112.15 | | 100.02 | | 113.48 | | 108.55 | | 7.42 | |
| 180 | 107.61 | | 109.85 | | 125.17 | | 114.21 | | 9.56 | |
| 200 | 115.52 | | 123.18 | | 123.13 | | 120.61 | | 4.41 | |
| 220 | 104.46 | | 113.12 | | 92.95 | | 103.51 | | 10.12 | |
| 240 | 93.21 | | 87.16 | | 113.27 | | 97.88 | | 13.67 | |
| 260 | 95.00 | | 89.42 | | 96.50 | | 93.64 | | 3.73 | |
| 280 | 117.28 | | 94.30 | | 92.47 | | 101.35 | | 13.83 | |
| 300 | 101.11 | | 111.05 | | 100.29 | | 104.15 | | 5.99 | |
| 320 | 104.33 | | 107.56 | | 119.16 | | 110.35 | | 7.80 | |
| 340 | 94.20 | | 98.00 | | 77.98 | | 90.06 | | 10.63 | |
| 360 | 72.53 | | 77.82 | | 74.53 | | 74.96 | | 2.67 | |
| 380 | 60.25 | | 63.26 | | 85.62 | | 69.71 | | 13.86 | |
|  |  |  | |  | |  | |  | |  |

**Data for Figure 10a**

| pH | Q1 | Q2 | Q3 | Q | SD |
| --- | --- | --- | --- | --- | --- |
| 1 | 5.25 | 4.33 | 3.47 | 4.35 | 0.73 |
| 2 | 21.48 | 12.58 | 9.68 | 14.58 | 5.02 |
| 3 | 144.02 | 190.03 | 130.02 | 154.69 | 25.63 |
| 4 | 795.10 | 879.97 | 688.95 | 788.01 | 78.14 |
| 5 | 982.89 | 991.51 | 785.01 | 919.80 | 95.38 |
| 6 | 1212.99 | 1319.00 | 1335.02 | 1289.00 | 54.15 |
| 7 | 1458.99 | 1131.24 | 1452.93 | 1347.72 | 153.09 |
| 8 | 717.85 | 838.01 | 706.15 | 754.00 | 59.59 |
| 9 | 758.71 | 680.51 | 799.98 | 746.40 | 49.54 |
| 10 | 680.97 | 699.96 | 635.34 | 672.09 | 27.12 |
| 11 | 379.92 | 463.61 | 432.36 | 425.30 | 34.53 |
| 12 | 286.86 | 295.90 | 279.82 | 287.53 | 6.58 |
| 13 | 101.30 | 99.32 | 115.76 | 105.46 | 7.33 |
| 14 | 67.65 | 70.43 | 76.10 | 71.39 | 3.52 |

**Data for Figure 10b**

**Data for p(AA-co-AM-co-MA)**

| Time | Q1 | Q2 | Q3 | Q | SD |
| --- | --- | --- | --- | --- | --- |
| 15 | 71.99 | 89.93 | 146.92 | 102.95 | 31.94 |
| 30 | 259.01 | 271.81 | 294.03 | 274.95 | 14.47 |
| 45 | 171.85 | 127.85 | 134.85 | 144.85 | 19.30 |
| 60 | 297.95 | 323.15 | 299.45 | 306.85 | 11.54 |
| 75 | 192.28 | 162.25 | 102.01 | 152.18 | 37.53 |
| 90 | 236.57 | 285.27 | 228.07 | 246.87 | 25.39 |
| 105 | 115.31 | 122.31 | 108.31 | 115.31 | 5.72 |

**Data for p(AA-co-AM)**

| Time | Q1 | Q2 | Q3 | Q | SD |
| --- | --- | --- | --- | --- | --- |
| 15 | 71.12 | 73.52 | 107.72 | 84.12 | 20.47 |
| 30 | 144.37 | 151.96 | 144.07 | 146.80 | 4.47 |
| 45 | 138.56 | 145.59 | 140.35 | 141.50 | 3.65 |
| 60 | 258.03 | 261.12 | 231.15 | 250.10 | 16.48 |
| 75 | 232.01 | 235.19 | 220.70 | 229.30 | 7.62 |
| 90 | 248.11 | 250.37 | 250.35 | 249.61 | 1.30 |
| 105 | 238.10 | 245.45 | 213.71 | 232.42 | 16.61 |

**Data for Figure 11a**

**Data for p(AA-co-AM-co-MA) in DW**

| T | time | Q1 | Q2 | Q3 | Q | SD |
| --- | --- | --- | --- | --- | --- | --- |
| 25 | 24 | 1540.06 | 1572.50 | 1480.50 | 1531.02 | 38.10 |
| 50 | 48 | 1209.75 | 1289.03 | 1391.91 | 1296.84 | 74.57 |
| 25 | 72 | 1741.10 | 1654.44 | 1797.64 | 1731.10 | 58.89 |
| 50 | 96 | 1432.77 | 1613.95 | 1401.93 | 1482.90 | 93.53 |
| 25 | 120 | 1680.54 | 1850.84 | 1851.65 | 1794.34 | 80.47 |
| 50 | 144 | 1568.13 | 1486.15 | 1618.14 | 1557.46 | 54.41 |

**Data for p(AA-co-AM) in DW**

| T | time | Q1 | Q2 | Q3 | Q | SD |
| --- | --- | --- | --- | --- | --- | --- |
| 25 | 24 | 852.35 | 849.95 | 898.16 | 866.82 | 27.17 |
| 50 | 48 | 855.02 | 847.32 | 858.70 | 853.68 | 5.81 |
| 25 | 72 | 817.04 | 832.24 | 836.64 | 828.64 | 10.28 |
| 50 | 96 | 802.42 | 825.04 | 810.10 | 812.52 | 11.50 |
| 25 | 120 | 670.20 | 650.11 | 671.78 | 664.03 | 12.08 |
| 50 | 144 | 381.20 | 377.15 | 397.13 | 358.16 | 10.56 |

**Data for Figure 11b**

**Data for p(AA-co-AM-co-MA) in SCS**

| T | time | Q1 | Q2 | Q3 | Q | SD |
| --- | --- | --- | --- | --- | --- | --- |
| 25 | 24 | 90.00 | 88.5 | 95.35 | 91.30 | 2.94 |
| 50 | 48 | 80.54 | 80.00 | 86.12 | 82.22 | 2.76 |
| 25 | 72 | 93.07 | 92.77 | 84.84 | 90.23 | 3.81 |
| 50 | 96 | 78.09 | 83.37 | 77.82 | 79.76 | 2.55 |
| 25 | 120 | 93.99 | 91.63 | 89.04 | 91.55 | 2.88 |
| 50 | 144 | 75.09 | 80.77 | 73.16 | 76.34 | 3.23 |

**Data for p(AA-co-AM) in SCS**

| T | time | Q1 | Q2 | Q3 | Q | SD |
| --- | --- | --- | --- | --- | --- | --- |
| 25 | 24 | 70.25 | 77.35 | 76.77 | 74.79 | 3.94 |
| 50 | 48 | 68.35 | 75.41 | 69.84 | 71.20 | 3.72 |
| 25 | 72 | 65.20 | 69.30 | 58.31 | 64.27 | 5.55 |
| 50 | 96 | 61.56 | 59.71 | 68.69 | 63.32 | 4.74 |
| 25 | 120 | 54.24 | 61.25 | 59.11 | 58.20 | 3.59 |
| 50 | 144 | 54.12 | 59.20 | 54.68 | 56.00 | 2.79 |
